# Performance of Greek Public Hospitals Before and After the Economic Recession and the Pandemic: Application of a Novel Cost Malmquist Index for Comparing Productivity Across Multiple Groups

**DOI:** 10.3390/healthcare13111253

**Published:** 2025-05-26

**Authors:** Argyro Fourlopoulou, Panos Xenos, George Messinios, Nikolaos Maniadakis

**Affiliations:** 1 Department of Public Health Policy, University of West Attica, 196 Alexandras Avenue, 11521 Athens, Greece; nmaniadakis@uniwa.gr; 2 Department of Statistics and Insurance Science, University of Piraeus, 80 Karaoli and Demetriou Str., 18534 Piraeus, Greece; pxenos@unipi.gr (P.X.); gmessinios01@gmail.com (G.M.)

**Keywords:** data envelopment analysis, cost Malmquist index, productivity measurement, productivity comparison, Greek public hospitals, group performance, Greek economic recession, COVID-19

## Abstract

**Background/Objectives**: This study introduces the *Multi Group Cost Malmquist Index* ($CMg_{m}$), a novel tool for comparing and ranking the cost efficiency of multiple groups of similar decision-making units operating in different contexts. It was applied to Greek public hospitals to assess productivity change between 2009 and 2021, covering the period before the economic recession and after the second lockdown during the COVID-19 pandemic. The study aimed to determine the impact of these external shocks on hospital efficiency and to identify differences in cost productivity based on hospital size and regional location. **Methods**: Data envelopment analysis was employed to compute the Malmquist indices for productivity change and ranking. Overall, 109 Greek public hospitals were analysed using three models: as a single group, classified by bed capacity, and classified by regional health authority (RHA). Cost productivity was decomposed into its core measures. **Results**: During the economic crisis, hospitals improved their cost productivity by 13.2%, whereas during the pandemic, it declined by 32.1%, primarily due to cost frontier deterioration resulting from increased healthcare demand and strained resources. Medium-sized hospitals exhibited higher cost efficiency than small and large hospitals. Regional disparities were also observed, with hospitals in the 5^th^ and 7^th^ RHAs outperforming those in 1^st^ and 2^nd^ RHAs. **Conclusions**: The findings highlight the pandemic’s disruptive impact on hospital cost productivity compared to the efficiency gains during the economic crisis. It is encouraging, though, that hospitals are recovering again after the lifting of strict lockdown measures. The $CMg_{m}$ is a valuable tool for policymakers, offering insights into hospital performance across multiple groups. Future healthcare policies should prioritise resource optimisation and address regional disparities to enhance system-wide efficiency and resilience in times of crisis.

## 1. Introduction

Productivity in public hospitals is a critical metric for evaluating how effectively health systems utilize their resources to deliver care. It reflects the relationship between inputs (such as labour, medical equipment, beds, and funding) and outputs (such as patient discharges, surgeries, or improved health outcomes). In an era of rising healthcare costs and growing demand, improving productivity is essential to ensure sustainability, equity, and quality in healthcare delivery.

Worldwide, public hospitals face intense pressure to do more with less. Populations are aging, chronic diseases are increasing, and medical technologies are advancing rapidly—often at high cost. At the same time, governments and health agencies are tasked with maintaining or improving service levels while controlling public spending. In this context, enhancing productivity allows hospitals to increase output or improve quality without proportionally increasing resources, thereby improving efficiency and access.

International organizations such as the WHO and OECD have repeatedly emphasized hospital productivity as a key dimension of health system performance. Efficient hospitals are better able to reduce wait times, avoid unnecessary procedures, and improve patient outcomes, all while staying within budgetary constraints.

In Greece, the issue of productivity in public hospitals gained particular importance during the economic crisis and the COVID-19 pandemic. Greece experienced nearly eight years of economic recession (2010–2018), followed by two years of a healthcare crisis due to the COVID-19 pandemic (2020–2021), placing immense pressure on the National Health System (NHS). To enhance efficiency and productivity, major reforms were introduced across the public healthcare sector.

During the economic crisis, Greece received three financial assistance packages from the European Commission, the European Central Bank, and the International Monetary Fund (the Troika)—the first in May 2010, the second in March 2012, and the third in July 2015—amounting to a total of EUR 337.21 billion. The Troika oversaw the implementation of these economic adjustment programmes until their conclusion in August 2018.

The healthcare sector was among the first to undergo substantial fiscal and financial reforms. The primary objectives of the reform agenda included the following:

(i) *Reducing expenditure* on pharmaceuticals, diagnostics, and publicly reimbursed healthcare services: key reforms aimed at this objective included the introduction of an electronic prescription system, the revision of the reimbursed drug list, the promotion of generic medicines, and the implementation of a clawback mechanism, among others;
(ii) *Improving hospital management and the overall efficiency* of the hospital sector: major initiatives included the establishment of the National Organisation for the Provision of Health Services (EOPYY), the enhancement of health information systems and modern accounting practices, the introduction of performance indicators, and the creation of a centralized procurement system for healthcare goods and services;
(iii) *Enhancing equity, efficiency, effectiveness, and governance* of the healthcare system: reforms under this objective included the harmonization of contributions and the introduction of standardized benefit packages, the consolidation of healthcare funds and entities, and the rollout of a primary healthcare network featuring a family doctor system;
(iv) *Ensuring universal health coverage*: this involved reforms such as expanding access to pharmaceuticals, diagnostic tests, and hospital inpatient care for the uninsured and low-income populations as well as reducing waiting times and improving transparency in the management of waiting lists [1,2,3,4,5,6,7,8,9,10,11,12,13].

Shortly after Greece exited the economic adjustment programmes, the COVID-19 pandemic emerged, with the first confirmed case on 26 February 2020, disrupting the NHS. One of the primary response measures was the efficient and productive reallocation of resources and recruitment of personnel. Policies and actions taken by the Greek government were summarised in Ladi et al. [14] and in Lampropoulou [15], while Sotiropoulos et al. [16] assessed their implementation. A detailed description of the government’s response measures can be found in Economou et al. [17].

The pandemic’s progression can be divided into two main phases, with the lockdowns serving as key milestones [17]:

*First Lockdown* (*March 2020–May 2020*): In response to the initial emergence of COVID-19, the Greek government swiftly implemented a nationwide lockdown in March 2020. These strict containment measures included school and business closures, travel restrictions, and a curfew. According to early epidemiological data, these actions were effective in limiting viral transmission and maintaining relatively low infection and mortality rates compared to other European countries during the first wave [18]. The health system remained largely unburdened, as case numbers were kept within manageable levels.

*Second Lockdown* (*November 2020–May 2021*): The second pandemic wave in autumn 2020 saw a significant rise in COVID-19 cases, hospitalizations, and intensive care unit (ICU) admissions, especially in Northern Greece. In November 2020, the government reintroduced strict lockdown measures, which remained in place—with intermittent adjustments—until May 2021. Despite the prolonged nature of this second lockdown, the healthcare system faced considerable strain, revealing systemic issues such as hospital overcrowding, limited ICU capacity, and regional disparities in healthcare resources [17,19].

From May 2021 onwards, no further nationwide lockdowns were imposed. Instead, targeted measures were implemented, such as mask mandates, restrictions for unvaccinated individuals, and regulations for indoor spaces, which continue to be adjusted based on prevailing conditions [17]. Thus, May 2021 can be considered a milestone marking the end of strict restrictions and the transition to a new phase of pandemic management.

Both lockdown phases had profound implications on the Greek NHS. While the first phase demonstrated the value of early intervention and public compliance, the second highlighted the vulnerability of the system under sustained pressure. The pandemic exposed long-standing weaknesses in the public health infrastructure, including underinvestment, staff shortages, and the absence of a robust primary healthcare network [17].

As the economic recession and the COVID-19 pandemic in Greece led to significant reforms across healthcare sector [14,15,20,21], it is essential from a health policy perspective to evaluate the impact of these reforms on hospital productivity over time. The ability to distinguish internal inefficiencies within a group of decision-making units (DMUs) from inefficiencies attributable to external factors, such as policies and environmental conditions, is well established through the development of two Malmquist indices: the *Group Malmquist Index* (Mg) [22] and the *Group Cost Malmquist Index* (CMg) [23]. These indices compare the productivity of two groups of similar DMUs operating in different contexts, with the former measuring input efficiency and the latter assessing cost efficiency.

The Mg was further refined into the *Multi Group Malmquist Index* ($Mg_{m}$) [22], which enables the comparison of more than two groups and ranks them in order of best to worst productivity in input terms.

This study introduces the *Multi Group Cost Malmquist Index* ($CMg_{m}$), as a methodological advancement for evaluating and ranking cost efficiency across multiple groups of DMUs. Building upon the original CMg framework, the $CMg_{m}$ facilitates a more granular and systematic assessment by incorporating inter-group comparisons. This enhancement enables a comprehensive examination of cost productivity dynamics, particularly within healthcare systems experiencing substantial external shocks. The applicability of this novel index is demonstrated using real-world data from the Greek public healthcare sector.

Furthermore, hospital performance was measured using the *Cost Malmquist Index* (CM) [24,25] to assess cost productivity changes. The CM, derived from the *Malmquist Index* (MI) [26], evaluates input–input price–output relationships while maintaining the advantages of MI, including ease of computation, minimal assumptions, and multiple decompositions of productivity change into its root causes. A key feature of CM is its ability to compute the *Allocative Efficiency Change Index* (AEC), which measures a DMU’s ability to optimise its input mix in accordance with prevailing input prices. More details on the usefulness and the computation of AEC can be found in [24,25].

The indices were computed using the non-parametric data envelopment analysis (DEA) method, a widely used approach in the healthcare industry for efficiency measurement [27]. DEA’s advantages include its ability to handle multi-input and multi-output technologies, its applicability to small datasets with an appropriate input/output ratio, and its minimal reliance on restrictive assumptions.

In summary, this study has two primary objectives:

1. To introduce the $CMg_{m}$ for comparing and ranking more than two groups of DMUs based on their cost efficiency;
2. To illustrate its applicability by analysing the productivity of 109 Greek public hospitals covering the period before the economic recession and after the second lockdown during the pandemic (2009–2021); the hospital sample was further classified by bed capacity and geographical region.

The structure of the paper is as follows: Section 2 outlines the methodology applied to Greek public hospitals, giving details on the data and sampling; the theoretical background on Malmquist indices, leading to the development of the novel Malmquist index for group cost productivity comparisons; former applications of these indices according to literature; the calculation of the values using DEA; and the three models of analysis. Section 3 analyses the results, while Section 4 and Section 5 discuss the findings of the study and present conclusions and limitations, respectively.

## 2. Methodology

### 2.1. Sample and Data

The sample comprises 109 Greek non-profit general public hospitals providing a full range of secondary and tertiary healthcare services. Specialty hospitals (psychiatric, oncology, etc.) were excluded. Further details on the sample are provided in Table A1 in Appendix A. The timeframe spans from 2009 to 2021, covering the economic recession (2009–2019) and the pandemic (2019–2021). A full description of the inputs–input prices–outputs bundle is presented in Table 1. The indicators that were chosen are among those most used in the literature, and they are appropriate for the scope of this particular analysis. The dataset was retrieved from the electronic platforms of the Greek Ministry of Health (MoH), namely esy.net (2012–2014) and BI forms (2015–2021). No data point was left empty. Every value was either recorded or estimated (interpolated). For the missing values, their estimates were based on existing values from previous or subsequent years. The dataset comprised a total of 16,015 cells, of which only 229 contained missing values, representing approximately 1.4% of the data. Given the minimal proportion of missingness, a sensitivity analysis was not considered necessary, as such a low level of imputation is unlikely to exert a meaningful influence on the results.

### 2.2. Productivity Indices

For the purpose of this study, as mentioned earlier, the following indices were used: CM for cost productivity change measurement and $CMg_{m}$ for cost productivity comparison. Additionally, *Cost Efficiency* (CE) *or Overall Efficiency Index* (OE) was measured along with its decompositions: *Technical Efficiency Index* (TE)*, Pure Technical Efficiency Index* (PTE)*, Allocative Efficiency Index* (AE)*,* and *Scale Efficiency Index* (SE).

#### 2.2.1. Cost Malmquist Index (CM)

The CM was developed by Maniadakis and Thanassoulis [24,25] to measure cost productivity changes. It is an adaptation of the MI [26] and incorporates decompositions such as the *Technical Efficiency Change Index* (TEC) and the *Technical Change Index* (TC) [28,29,30,31,32]. The CM integrates input prices alongside input quantities and is applicable in contexts where producers are assumed to be cost minimisers, provided input–output quantities and price data are available.

The CM evaluates productivity change in cost terms by comparing cost frontiers over two periods, $t_{0}$ and $t_{1}$. A value below 1 indicates progress, a value above 1 denotes regression, and a value of 1 signifies constant productivity. CM is further decomposed into the *Overall Efficiency Change Index* (OEC) and the *Cost Technical Change Index* (CTC). These components can be further broken down as follows:

- OEC is divided into the *Technical Efficiency Change* (TEC) and *Allocative Efficiency Change* (AEC) indices. The AEC, a key feature of CM, assesses a DMU’s ability to optimise its input mix according to prevailing input prices;
- CTC is further decomposed into the *Technical Change Index* (TC) and the *Price Effect Index* (PE), which captures the influence of relative input price changes on minimum production costs.

The mathematical expressions for calculating the above indices are presented below:

(1) $$CM=\sqrt{\frac{CE_{\left(P^{t_{0}}I^{t_{0}}\right)}}{CE_{\left(P^{t_{1}}I^{t_{0}}\right)}}\frac{CE_{\left(P^{t_{0}}I^{t_{1}}\right)}}{CE_{\left(P^{t_{1}}I^{t_{1}}\right)}}}$$

(2) $$CTC=\sqrt{\frac{CE_{\left(P^{t_{1}}I^{t_{1}}\right)}}{CE_{\left(P^{t_{1}}I^{t_{0}}\right)}}\frac{CE_{\left(P^{t_{0}}I^{t_{1}}\right)}}{CE_{\left(P^{t_{0}}I^{t_{0}}\right)}}}$$

(3) $$OEC=\frac{CE_{\left(P^{t_{0}}I^{t_{0}}\right)}}{CE_{\left(P^{t_{1}}I^{t_{1}}\right)}}$$

(4) $$TC=\sqrt{\frac{E_{\left(P^{t_{1}}F^{t_{1}}\right)}}{E_{\left(P^{t_{1}}F^{t_{0}}\right)}}\frac{E_{\left(P^{t_{0}}F^{t_{1}}\right)}}{E_{\left(P^{t_{0}}F^{t_{0}}\right)}}}$$

(5) $$TEC=\frac{E_{\left(P^{t_{0}}F^{t_{0}}\right)}}{E_{\left(P^{t_{1}}F^{t_{1}}\right)}}$$

(6) $$AEC=\frac{CE_{\left(P^{t_{0}}I^{t_{0}}\right)}E_{\left(P^{t_{1}}F^{t_{1}}\right)}}{CE_{\left(P^{t_{1}}I^{t_{1}}\right)}E_{\left(P^{t_{0}}F^{t_{0}}\right)}}$$

(7) $$PE=\sqrt{\frac{CE_{\left(P^{t_{1}}I^{t_{1}}\right)}}{CE_{\left(P^{t_{1}}I^{t_{0}}\right)}}\frac{CE_{\left(P^{t_{0}}I^{t_{1}}\right)}}{CE_{\left(P^{t_{0}}I^{t_{0}}\right)}}\frac{E_{\left(P^{t_{1}}F^{t_{0}}\right)}}{E_{\left(P^{t_{1}}F^{t_{1}}\right)}}\frac{E_{\left(P^{t_{0}}F^{t_{0}}\right)}}{E_{\left(P^{t_{0}}F^{t_{1}}\right)}}}$$

where CE: cost efficiency; E: efficiency; $P^{t_{0}}=\left(x^{t_{0}},y^{t_{0}},w^{t_{0}}\right):$ all input $x^{t_{0}}$/output $y^{t_{0}}$/input prices $w^{t_{0}}$ combinations of DMUs in period $t_{0}$; $P^{t_{1}}=\left(x^{t_{1}},y^{t_{1}},w^{t_{1}}\right):$ all input $x^{t_{1}}$/output $y^{t_{1}}$/input prices $w^{t_{1}}$ combinations of DMUs in period $t_{1}$; $I^{t_{0}}$, $I^{t_{1}}$: iso-cost frontiers in periods $t_{0}$ and $t_{1}$, respectively; $F^{t_{0}}$, $F^{t_{1}}$: technical frontiers in periods $t_{0}$ and $t_{1}$, respectively.

The functional relationships among the aforementioned equations are as follows:

(8) CM=OEC×CTC  =TEC×AEC×TC×PE  =MI×AEC×PE

For further details on CM computation, see Maniadakis and Thanassoulis [24,25].

#### 2.2.2. Group Cost Malmquist Index (CMg)

The CMg, developed by Thanassoulis et al. [23], integrates the concepts of Mg and CM. The Mg, originally introduced by Camanho and Dyson [22], allows comparison of productivity between two groups of DMUs operating in different contexts (e.g., group A and group B). Unlike Mg, which only considers inputs, CMg incorporates input prices, making it suitable for cost-efficiency analysis.

CMg measures the geometric mean of the cost efficiency ratios of DMUs in group B and group A relative to the cost frontier of group A and vice versa, and each DMU has its own input prices. It is decomposed into the following:

- *Group Overall Efficiency Spread Index* (OESg), which is further divided into *Group Technical Efficiency Spread Index* (TESg) and *Group Allocative Efficiency Index* (AEg);
- *Group Cost Frontier Gap Index* (CFg), which is further divided into *Group Technical Frontier Gap Index* (TFg) and *Group Price Effect Index* (PEg).

A CMg value greater than 1 indicates that DMUs in group B are more cost-efficient than those in group A. A value below 1 suggests the opposite, while a value of 1 indicates equal cost productivity between the two groups. Further details on CMg and its components can be found in Thanassoulis et al. [23].

#### 2.2.3. Multi Group Malmquist Index ($Mg_{m}$)

In cases where more than two groups are involved, Camanho and Dyson [22] introduced the *Multi Group Malmquist Index* ($Mg_{m}$) by modifying the TFg into the *Multi Group Technical Frontier Gap Index* ($TFg_{m}$) to satisfy the circular relationship. The TESg remains unchanged in both cases. The mathematical expressions for calculating the above indices are presented below:

(9) $$TFg_{m}={\left[\overset{N}{\underset{1}{\prod }}\frac{{\left[\prod_{1}^{\delta_{i}}E_{\left(P^{i}F^{A}\right)}\right]}^{1/\delta_{i}}}{{\left[\prod_{1}^{\delta_{i}}E_{\left(P^{i}F^{B}\right)}\right]}^{1/\delta_{i}}}\right]}^{1/N}$$

(10) $$TESg=\frac{{\left[\prod_{1}^{\delta_{B}}E_{\left(P^{B}F^{B}\right)}\right]}^{1/\delta_{B}}}{{\left[\prod_{1}^{\delta_{A}}E_{\left(P^{A}F^{A}\right)}\right]}^{1/\delta_{A}}}$$

(11) $$Mg_{m}=TFg_{m}\times TESg$$

where E: efficiency; i: groups $\left(i=1,\ldots ,N\right)$; N: number of groups; $\delta_{i},\delta_{A},\delta_{B}:$ number of DMUs of group i, A, and B, respectively; $P^{i}=\left(x^{i},y^{i}\right):$ all input $x^{i}$-output $y^{i}$ combinations of $\delta_{i}$ DMUs of group i; $P^{A}=\left(x^{A}y^{A}\right):$ all input $x^{A}$-output $y^{A}$ combinations of $\delta_{A}$ DMUs of group A; $P^{B}=\left(x^{B}y^{B}\right):$ all input $x^{B}$-output $y^{B}$ combinations of $\delta_{B}$ DMUs of group B; $F^{A},F^{B}:$ technical frontiers of group A and group B, respectively.

Further details for the computation and interpretation of $Mg_{m}$ and its components can be found in Camanho and Dyson [22].

#### 2.2.4. Multi Group Cost Malmquist Index ($CMg_{m}$)

This study extends CMg by ensuring that it satisfies the circular relationship when comparing more than two groups. Similar to $Mg_{m}$, CMg is adjusted to $CMg_{m}$ by modifying the CFg into the *Multi Group Cost Frontier Gap Index* ($CFg_{m}$) and the PEg into the *Multi Group Price Effect Index* ($PEg_{m}$). OESg remains unchanged in this transformation. $CMg_{m}$ enables ranking of multiple groups by cost efficiency, which is the key contribution of this extended Malmquist index.

The mathematical expressions for calculating the above indices are presented below:

(12) $$OESg=\frac{{\left[\prod_{j=1}^{\delta_{B}}CE_{\left(P^{B}I^{B}\right)}\right]}^{\frac{1}{\delta_{B}}}}{\;{\left[\prod_{j=1}^{\delta_{A}}CE_{\left(P^{A}I^{A}\right)}\right]}^{\frac{1}{\delta_{A}}}}$$

(13) $$AEg=\frac{{\left[\prod_{j=1}^{\delta_{B}}CE_{\left(P^{B}I^{B}\right)}\right]}^{\frac{1}{\delta_{B}}}}{{\left[\prod_{j=1}^{\delta_{A}}CE_{\left(P^{A}I^{A}\right)}\right]}^{\frac{1}{\delta_{A}}}}\times \frac{{\left[\prod_{j=1}^{\delta_{A}}E_{\left(P^{A}F^{A}\right)}\right]}^{\frac{1}{\delta_{A}}}}{{\left[\prod_{j=1}^{\delta_{B}}E_{\left(P^{B}F^{B}\right)}\right]}^{\frac{1}{\delta_{B}}}}$$

(14) $$CFg_{m}={\left[\overset{N}{\underset{1}{\prod }}\frac{{\left[\prod_{1}^{\delta_{i}}CE_{\left(P^{i}I^{A}\right)}\right]}^{1/\delta_{i}}}{{\left[\prod_{1}^{\delta_{i}}CE_{\left(P^{i}I^{B}\right)}\right]}^{1/\delta_{i}}}\right]}^{1/N}$$

(15) $$PEg_{m}=\frac{CFg_{m}}{TFg_{m}}$$

(16) $$CMg_{m}=OESg\times CFg_{m}$$

The correlation between these equations is as follows:

(17) $$\begin{array}{cc} CMg_{m} & \;=OESg\times CFg_{m}\;\;\;\;\;\;\;\;\;\;\;\;\;\;\;\;\;\;\;\;\;\;\;\;\;\;\;\;\;\\ & =TESg\times AEg\times TFg_{m}\times PEg_{m}\;\;\\ & =Mg_{m}\times AEg\times PEg_{m}\;\;\;\;\;\;\;\;\;\;\;\;\;\;\;\;\; \end{array}$$

### 2.3. Applications of the Productivity Indices

#### 2.3.1. Studies Based on *Cost Malmquist Index* (CM)

Maniadakis and Thanassoulis [25] proposed the CM and its decompositions, illustrating it with a sample of 30 Greek public hospitals for the period 1992–1993. To our knowledge, this paper is the only one to date to use CM to measure the performance of Greek public hospitals. In fact, no other study has applied this index to any other sector in Greece.

Earlier, Maniadakis and Thanassoulis [24] used their index to evaluate the performance of 75 Scottish acute hospitals following the introduction of reforms in the United Kingdom, covering the period 1991–1996. Asghar et al. [33] also applied CM in the healthcare sector, estimating the dynamics of cost productivity and its determinants within the healthcare systems of 55 member states of the Organisation of Islamic Cooperation over a five-year period (2011–2015).

Several studies have extended CM: Hosseinzadeh et al. [34] further analysed its two components, while Tohidi et al. [35] proposed the *global cost Malmquist productivity index*, which is circular and provides a single measure of productivity change.

Beyond the healthcare sector, CM has been applied in various industries, including agriculture, farming, air navigation, education, banking, tourism, water supply, insurance, forestry, biopharmaceuticals, security, and thermal power generation [36,37,38,39,40,41,42,43,44,45,46,47,48,49,50,51,52].

#### 2.3.2. Studies Based on *Group Cost Malmquist Index* (CMg)

Regarding CMg, only a few studies have utilised this index. Habibpoor et al. [53] applied it to assess the productivity of bank branches across different regions. Walheer [54] extended CMg to provide group-specific results for each output separately. His index is a different approach that enables identification of which output and in what percentage contributes to changes in cost performance. As an illustration, it is applied in more than two groups—five electricity plant districts—and, like CMg, this output-specific approach lacks the circularity property, too.

To address the circularity issue, Walheer [55] modified the CMg with the *global Malmquist* variation to incorporate its advantages. It is very well elaborated in his paper that one of the drawbacks of CMg is non-circularity. Thus, the main aim of our paper is the modification of CMg into the novel index of the *Multi Group Cost Malmquist Index* ($CMg_{m}$), which is circular and can compare more than two groups of DMUs. The two versions of indices for group cost performance comparison use different methodology in constructing the technical and cost frontiers. Therefore, the choice of methodology depends on the aspect in which the comparison is intended.

### 2.4. Calculation of the Productivity Indices

The non-parametric mathematical programming approach of input-oriented DEA was employed to calculate cost efficiencies. The basic DEA model was introduced by Farrell in [56], but its first application dates back to the 1980s, when it was developed by Charnes et al. [57] and later extended to incorporate variable returns to scale by Banker et al. [58]. Given the unique nature of healthcare markets, DEA is currently the most appropriate method for measuring efficiency in this sector [59].

Reviews on efficiency measurement methodologies have shown that 72% of studies apply the DEA approach [60]. Several studies have reviewed healthcare performance using DEA [61,62,63,64,65,66,67], while others have systematically examined its application in the healthcare sector [59,68,69].

Moreover, there is a substantial number of studies that have reviewed DEA applications in hospital efficiency measurement, including [70,71,72,73,74,75,76,77,78,79].

For a more comprehensive and up-to-date list of DEA-related articles across various sectors, see Emrouznejad and Yang [27] and Seiford [80]. The efficiencies and cost efficiencies were computed using the R programming language, specifically the “rDEA” package.

### 2.5. Models of Analysis

In the first model, all hospitals were treated as a single group, and OE and its decompositions were measured. For the same sample, cost productivity change and its components were also assessed using CM.

In the second model, hospitals were classified into three groups based on bed capacity. The cost productivity of each group was compared with the others annually from 2009 to 2021 using $CMg_{m}$. The three groups include the following:

1. Small-sized (S) hospitals with fewer than 200 beds;
2. Medium-sized (M) hospitals with 200–400 beds;
3. Large-sized (L) hospitals with more than 400 beds.

The grouping of hospitals based on bed capacity is a widely used and accepted approach in the literature [81,82,83,84,85,86,87,88,89,90]. This classification provides a simple yet effective way to account for scale-related differences in capacity and performance.

In the third model, hospitals were classified into seven groups according to their respective regional health authorities (RHAs) under the MoH. Each group was compared with the other six in terms of cost productivity over the same period using $CMg_{m}$. The groups are as follows:

1^st^ RHA of Attiki.

2^nd^ RHA of Piraeus and the Aegean.

3^rd^ RHA of Macedonia.

4^th^ RHA of Macedonia and Thrace.

5^th^ RHA of Thessaly and Sterea Ellada.

6^th^ RHA of Peloponnese, Ionian Islands, Epirus, and Western Greece.

7^th^ RHA of Crete.

More details on the composition of the hospital groups are provided in Table A1 in Appendix A.

## 3. Results

### 3.1. Entire Hospital Sample Analysis

The OE and its decompositions for the entire hospital sample from 2009 to 2021 are shown in Table 2.

Across this period, OE was 63.8%, indicating that hospitals could reduce operating costs by 36.2% to achieve full efficiency. Inefficiencies were primarily due to excessive input usage, as TE was 71.6%, meaning hospitals could reduce input levels by 28.4%. On the other hand, AE was relatively high (89%), suggesting that hospitals successfully optimised their input mix according to prevailing input prices. Additionally, hospitals operated close to the optimal scale, as indicated by the high SE level. This trend persisted during the economic crisis (2009–2019), whereas all efficiency metrics declined during the pandemic (2019–2021).

Table 3 presents CM and its components for each consecutive year, the economic crisis period (2009–2019), the pandemic (2019–2021), and the overall period (2009–2021).

The CM shows continuous cost productivity progress from 2010–2011 to 2014–2015, which was reversed in 2015–2016. The highest progress (9.5%) was observed in 2016–2017, followed by regression in 2018–2019. Interestingly, periods of regression coincided with election years (2015 and 2019), government changes, policy shifts, conflicts with the Troika, and the end of economic adjustment programmes. The worst regression (38.8%) occurred during the strict lockdown measures of the pandemic (2019–2020). After the second lockdown, the index exhibited an upward trend of 4.6% progression in 2020-2021.

For the duration of the economic crisis (2009–2019), CM indicates a 13.2% cost productivity improvement, whereas a 32.1% decline occurred during the pandemic (2019–2021). Overall, the CM for the full period (2009–2021) reveals a 15.6% regression.

The primary drivers of cost productivity changes are captured in CM components. From 2009 to 2019, the CTC advanced by 14.9%, mainly due to 14% progress in the technical frontier (TC), as PE progress was only 1%. This indicates that input prices (salaries and bed operational costs) changed marginally and had minimal impact on cost reductions. The second component, OEC, declined by 1.9%, meaning hospitals failed to improve efficiency through input reduction, as indicated by a 2.5% regression in TEC. However, a slight alignment with prevailing input prices (0.6% improvement in AEC) was observed.

During the pandemic, a 36.7% regression in CTC was the primary factor behind the overall decline in productivity. Paradoxically, hospitals improved their overall efficiency by 3.4%. Over the full period, CTC declined by 17.4%, while OEC showed a slight improvement of 1.5%. Table 4 details the number of hospitals that progressed or regressed according to CM. In most years, significantly more hospitals progressed than regressed. Notably, in 2009–2010, the first year of reform implementation, most hospitals regressed. However, by 2011, 80 hospitals showed an improvement. From then on, a steady upward trend was observed, with 2017 marking the highest number (83) of progressing hospitals. Unfortunately, this trend abruptly halted during the health crisis, with all 109 hospitals regressing in 2019–2020. Encouragingly, the results for 2020–2021 suggest a return to positive trends.

### 3.2. Bed Capacity Grouping Analysis

According to bed capacity, hospitals were categorised into three groups: S, M, and L. These groups were compared in dyads using $CMg_{m}$, generating three comparisons per year. The results are shown in Table A2 in the Appendix A. One group was used as a reference, meaning its index values were set to 1, allowing the other groups to be ranked based on performance. In this analysis, the reference group was S, leading the transformation of Table A2 into Table 5, with the ranking results according to $CMg_{m}$. The values of the remaining components follow a random order depending on the group.

The M group demonstrates superior cost productivity, ranking first for nine years. Conversely, the L group ranked third for six years and second for four years. In 2018, 2019, and 2020, the rankings were reversed, but in 2021, M regained the top position. The superior performance of M is attributed to its strong performance in OESg and TESg. Hospitals of the M group operate very close to their cost and technological frontiers, achieving their minimum attainable costs with minimal inputs, making them more efficient. In contrast, the S group exhibits the weakest performance in OESg and TESg. However, it ranked first every year in $CFg_{m}$ and $TFg_{m}$, indicating it possesses the best cost and technological frontiers, even though its hospitals operate at a distance from them.

Regarding AEg, there is no consistent leading group, as each has ranked first an equal number of times but in a random order: L for four years, M for five years, and S for four years. This suggests that hospitals across all groups occasionally manage to minimise costs by optimising their input mix and input prices. Regarding $PEg_{m}$, the L group ranked first for ten years, indicating that L hospitals have a lower potential for cost savings through input mix adjustments once they achieve technical efficiency within their own technological frontier, given the prevailing input prices.

### 3.3. Regional Grouping Analysis

Each group of the seven RHAs was compared with the others in dyads using $CMg_{m}$, generating 21 comparisons. The results are shown in Table A3 in Appendix A. The 1^st^ RHA of Attiki, serves as the reference group, meaning its index values are set to 1. Thus, the seven RHAs can be ranked from highest to lowest productivity, and Table A3 is transformed into Table 6 with the ranking results. The values of the remaining components follow a random order depending on the group.

Over the years, group rankings have varied. By assessing how frequently each group occupied a particular ranking, they can be classified as having either strong or weak performance. Based on $CMg_{m}$ and $Mg_{m}$ rankings, the 1^st^ RHA of Attiki and the 2^nd^ RHA of Piraeus and the Aegean exhibit the lowest productivity in cost and input terms, as they were ranked last most frequently. The 4^th^ RHA of Macedonia and Thrace and the 6^th^ RHA of Peloponnese, Ionian Islands, Epirus, and Western Greece display moderate performance, often occupying middle positions, whereas the 3^rd^ RHA of Macedonia consistently secures second place. The 5^th^ RHA of Thessaly and Sterea Ellada and the 7^th^ RHA of Crete demonstrate high performance, ranking first or second most frequently.

Examining $CMg_{m}$ components, hospitals in the 4^th^ RHA of Macedonia and Thrace and the 7^th^ RHA of Crete operate very close to their cost and technical frontiers. This means they perform at minimum attainable costs with the lowest input quantities, as reflected in their lower efficiency spread around cost and technical frontiers (higher OESg and TESg values). Conversely, hospitals in the 2^nd^ RHA of Piraeus and the Aegean exhibit the weakest cost and technical efficiency spread. The 6^th^ RHA of Peloponnese, Ionian Islands, Epirus, and Western Greece forms the most efficient cost and technical frontiers, ranking first in $CFg_{m}$ and $TFg_{m}$ in most years, whereas the 4^th^ RHA of Macedonia and Thrace appears to have the least efficient frontiers.

The 7^th^ RHA of Crete excels in selecting the optimal mix of inputs and input prices, ranking first most frequently in AEg. In contrast, the 2^nd^ RHA of Piraeus and the Aegean ranks last most years. Regarding cost-saving potential, no single group consistently outperformed, as all RHAs have occupied various ranks in $PEg_{m}$ over the years.

## 4. Discussion

This study introduces the $CMg_{m}$ as a novel tool for comparing and ranking the cost efficiency of multiple groups of DMUs, demonstrating its applicability in evaluating the performance of Greek public hospitals covering the period before the economic recession and after the second lockdown during the COVID-19 pandemic. By extending the original CMg*,* the $CMg_{m}$ provides a more nuanced analysis, accounting for the ranking of multiple groups and thus offering insights into cost productivity changes within a healthcare system subjected to major external shocks.

The findings highlight the substantial impact of the economic recession and pandemic on hospital cost productivity. Specifically, the Greek public hospitals demonstrated a significant decline in cost productivity during the pandemic (2019–2021), with a 32.1% decrease, which is in stark contrast to the positive productivity growth observed during the economic crisis (2009–2019). The cost frontier regression, which accounted for much of this decline, was driven by the sudden increase in demand for healthcare services, coupled with a strained resource base and the necessity for rapid resource reallocation during the health crisis. Despite the pandemic-related challenges, the results also revealed a slight recovery in 2021, after the gradual relaxation of the lockdown restrictions, suggesting that hospitals began adapting to the new realities of the pandemic, likely due to operational adjustments and government interventions.

The analysis of Greek hospitals based on bed capacity revealed that M hospitals consistently outperformed both S and L hospitals in terms of cost productivity. This performance can be attributed to the operational efficiency of M hospitals, which operate closer to their cost and technological frontiers, allowing them to optimize input use more effectively. On the other hand, S hospitals, while possessing the best cost and technological frontiers, struggled to achieve optimal efficiency, possibly due to their limited scale and higher per-unit costs. L hospitals, while showing strong performance in certain areas such as price efficiency, often lagged behind in technical efficiency, which contributed to their relatively lower cost productivity rankings.

The regional analysis provided additional insights into the geographic disparities in hospital performance across Greece. The results demonstrated a clear distinction in productivity across the seven RHAs. Specifically, the 1^st^ RHA of Attiki and the 2^nd^ RHA of Piraeus and the Aegean exhibited the lowest productivity, while the 5^th^ RHA of Thessaly and Sterea Ellada and the 7^th^ of Crete demonstrated the highest productivity levels. These findings suggest that geographical factors, such as local healthcare policies, resource distribution, and administrative efficiency, may significantly influence hospital performance. Hospitals in certain regions were able to perform at minimum attainable costs with optimized input quantities, which aligns with findings from other studies highlighting regional variations in healthcare delivery efficiency.

The use of the $CMg_{m}$ allowed for an in-depth comparison of cost productivity across multiple groups, providing a more granular understanding of hospital performance. The ability to rank multiple groups based on cost efficiency while accounting for the effects of external factors such as economic crises and pandemics makes the $CMg_{m}$ a powerful tool for health policy decisionmakers. It provides critical insights into the relative efficiency of healthcare systems in different regions and across different hospital sizes, allowing policymakers to target reforms more effectively.

In practical terms, the study’s results can support health authorities and hospital managers in identifying the most efficient hospital types and regions, enabling the reallocation of resources toward high-performing units and the implementation of efficiency-enhancing interventions in underperforming areas. Moreover, the $CMg_{m}$ can be applied periodically as a benchmarking mechanism to monitor the evolution of hospital efficiency over time and guide targeted management decisions.

Based on the findings, several policy actions can be proposed to improve the dynamics of the Greek public health sector. First, health policy should incentivize the replication of successful operational practices from M-sized hospitals across the system, particularly in terms of input optimization. Second, resource reallocation strategies should be tailored geographically, prioritizing support to RHAs with persistently low productivity. Third, national-level interventions should focus on reducing technical inefficiencies in L hospitals, possibly through the modernization of administrative processes and investment in workforce training. Lastly, establishing a real-time hospital productivity monitoring system, grounded on tools like $CMg_{m}$, could significantly enhance transparency, accountability, and data-driven policymaking in the healthcare sector.

## 5. Conclusions

This study extends the Malmquist index framework by introducing the $CMg_{m}$ to evaluate the cost productivity of multiple groups within a healthcare system, exemplified by Greek public hospitals during the economic recession and the COVID-19 pandemic. The findings underscore the critical role of external factors such as economic crises and health emergencies in shaping hospital productivity. The results indicate that while the reforms during the economic recession (2009–2019) led to improvements in cost productivity, the pandemic (2019–2021) resulted in significant regressions, underscoring the vulnerabilities of healthcare systems during times of crisis. Nevertheless, the outcomes are encouraging, as they suggest a recovery following the definitive end of the lockdowns in May 2021.

The grouping analysis based on hospital size and regional location revealed significant variations in cost productivity. M hospitals emerged as the most efficient, while S and L hospitals faced distinct challenges. Additionally, geographical disparities in hospital performance suggest that regional factors such as governance and resource allocation play an important role in shaping healthcare efficiency.

The limitations of this study primarily stem from the weaknesses of the DEA method. It tends to attribute all deviations from the frontier to inefficiency and often estimates an excessive number of DMUs as efficient. To address this issue, we dedicated significant effort to curate the datasets used. There were instances where the data in the database were either incorrect or absent, so direct contact with the hospitals was made, or data were interpolated where archives were missing. Furthermore, special care was taken to ensure the comparability of the data. Each variable comprises several subcategories, and every entry was checked for consistency. For example, surgeries are classified as minor, medium, and major severity, so it was ensured that the number of surgeries reported by all hospitals included all three subcategories. Additionally, the dataset did not include quality variables, which would make the productivity measurement more concrete. Unfortunately, this is not particularly plausible given the limited formal registration of quality variables. Data such as QUALYs, survival rates, and mortality rates are not yet consistently recorded across all hospitals, nor do they adhere to any standardisation. One example of an attempt to assess quality in efficiency measurement in Greek hospitals is the study by Xenos et al. [91], which encountered the same limitations. A potential update to the current BI forms database could involve expanding it to include quality data as well.

Overall, the $CMg_{m}$ provides a valuable tool for comparing the cost productivity of healthcare systems, especially in times of reform and crisis. The study highlights the need for tailored policy interventions that consider the unique challenges faced by hospitals of different sizes and regions. As healthcare systems continue to grapple with the aftermath of the pandemic and future health challenges, tools like the $CMg_{m}$ will be essential for guiding evidence-based policy decisions aimed at improving healthcare efficiency and sustainability.

Future research could involve applying the $CMg_{m}$ in other economic sectors or testing it in a comparative framework against other methodologies focused on cost effectiveness and productivity measurement. Such explorations could enhance the methodological robustness and applicability of $CMg_{m}$ across broader contexts.

## Figures and Tables

**Table 1:** Description of inputs, input prices, and outputs.

Indicator	Description	Time of Measurement
INPUTS		
Bed Capacity	Number of fully equipped operational beds	In December each year
Total Personnel	Total number of Doctors: Specialised consultants and interns. Nurses: Registered nurses with higher education (four-year courses). Scientific staff: Physiotherapists, pharmacists, dentists, radiologists, dieticians, psychologists, biochemists, and social workers. Clinical support staff: Vocational nurses, laboratory staff, and others. Administrative staff: Economists, accountants, secretaries, statisticians, and IT staff. Technical staff: electricians, plumbers, builders, and general maintenance workers.	
INPUT PRICES		
Operating Costs per Bed Capacity	Pharmaceutical costs Consumables costs Services costs	Total for the 12 months of each year
Salaries Cost per Total Personnel		
OUTPUTS		
Hospitalised Patients	Total number of patients admitted to all hospital sectors.	Total for the 12 months of each year
Outpatient Cases	Total number of patients who visited the all-day outpatient department and the emergency department.	
Operations	Number of scheduled or emergency surgical procedures categorised as minor, moderate, or major severity.	

**Table 2:** Overall Efficiency of 109 Greek public hospitals and decompositions during 2009–2021.

Year	*OE*	*TE*	*PTE*	*AE*	*SE*
2009	0.609	0.695	0.790	0.876	0.880
2010	0.707	0.764	0.819	0.925	0.933
2011	0.715	0.785	0.839	0.911	0.936
2012	0.609	0.695	0.790	0.876	0.880
2013	0.683	0.768	0.815	0.889	0.942
2014	0.706	0.779	0.823	0.905	0.947
2015	0.687	0.770	0.826	0.893	0.932
2016	0.599	0.707	0.766	0.847	0.923
2017	0.610	0.675	0.746	0.904	0.905
2018	0.559	0.624	0.730	0.896	0.854
2019	0.597	0.664	0.750	0.899	0.886
2020	0.616	0.707	0.760	0.871	0.930
2021	0.618	0.697	0.743	0.886	0.938
MEAN 2009–2019	0.641	0.719	0.790	0.893	0.910
MEAN 2019–2021	0.610	0.689	0.751	0.886	0.918
MEAN 2009–2021	0.638	0.716	0.784	0.890	0.914

*OE*: Overall Efficiency; *TE*: Technical Efficiency; *PTE*: Pure Technical Efficiency; *AE*: Allocative Efficiency; *SE*: Scale Efficiency.

**Table 3:** Cost productivity change of 109 Greek public hospitals with *CM* and decompositions during 2009–2021.

Periods	*CM*	*CTC*	*OEC*	*AEC*	*PE*	*MI*	*TC*	*TEC*
2009–2010	1.007	1.170	0.861	0.975	1.028	1.005	1.138	0.883
2010–2011	0.955	0.967	0.988	1.015	0.980	0.960	0.986	0.973
2011–2012	0.990	0.937	1.057	0.995	0.989	1.007	0.947	1.063
2012–2013	0.971	0.979	0.991	1.008	0.983	0.979	0.996	0.984
2013–2014	0.978	1.011	0.967	1.005	0.994	0.979	1.017	0.962
2014–2015	0.993	0.967	1.027	1.002	0.991	1.000	0.976	1.025
2015–2016	1.040	0.907	1.147	1.021	0.979	1.041	0.926	1.124
2016–2017	0.905	0.921	0.982	0.973	1.025	0.907	0.899	1.009
2017–2018	0.983	0.901	1.091	0.994	1.007	0.981	0.894	1.097
2018–2019	1.024	1.093	0.936	1.007	0.993	1.024	1.101	0.930
2019–2020	1.388	1.431	0.970	0.983	1.030	1.371	1.389	0.987
2020–2021	0.954	0.958	0.996	1.018	0.981	0.956	0.977	0.978
**2009–2019**	**0.868**	**0.851**	**1.019**	**0.994**	**0.990**	**0.882**	**0.860**	**1.025**
Mean 2009–2019	0.984	0.982	1.002	0.999	0.997	0.988	0.985	1.003
**2019–2021**	**1.321**	**1.367**	**0.966**	**1.001**	**0.993**	**1.329**	**1.377**	**0.965**
Mean 2019–2021	1.151	1.171	0.983	1.001	1.005	1.144	1.165	0.983
**2009–2021**	**1.156**	**1.174**	**0.985**	**0.995**	**0.993**	**1.170**	**1.182**	**0.990**
Mean 2009–2021	1.010	1.011	0.999	1.000	0.998	1.012	1.013	0.999

*CM*: Cost Malmquist Index; *CTC*: Cost Technical Change; *OEC*: Overall Efficiency Change; *AEC*: Allocative Efficiency Change; *PE*: Price Effect; *MI*: Malmquist Index; *TC*: Technical Change; *TEC*: Technical Efficiency Change.

**Table 4:** Number of Greek public hospitals that progressed or regressed according to *CM* during 2009–2021.

	2009– 2010	2010– 2011	2011– 2012	2012– 2013	2013– 2014	2014– 2015	2015– 2016	2016– 2017	2017– 2018	2018– 2019	2019– 2020	2020– 2021	2009– 2019	2019– 2021	2009– 2021
**<1**	47	80	62	73	71	56	44	83	68	37	0	67	81	6	34
**>1**	62	29	47	35	37	53	65	26	40	72	109	42	28	103	75

<1: progress; >1: regress; *CM*: Cost Malmquist Index.

**Table 5:** Ranking of groups of hospitals per bed capacity according to *CMg_m_* and components during 2009–2021.

Year	Ranking of Groups		*CMg_m_*	*OESg*	*CFg_m_*	*AEg*	*PEg_m_*	*Mg_m_*	*TESg*	*TFg_m_*
2009	1st	M	1.043	1.364	0.765	1.049	0.957	1.040	1.300	0.800
	2nd	S	1.000	1.000	1.000	1.000	1.000	1.000	1.000	1.000
	3rd	L	0.983	1.354	0.726	1.055	0.982	0.949	1.284	0.739
2010	1st	S	1.000	1.000	1.000	1.000	1.000	1.000	1.000	1.000
	2nd	M	0.995	1.147	0.867	1.015	0.992	0.988	1.131	0.874
	3rd	L	0.967	1.051	0.920	1.000	1.048	0.923	1.051	0.878
2011	1st	M	1.083	1.116	0.970	1.012	1.016	1.053	1.103	0.955
	2nd	S	1.000	1.000	1.000	1.000	1.000	1.000	1.000	1.000
	3rd	L	0.959	1.076	0.891	1.014	1.050	0.901	1.061	0.849
2012	1st	M	1.119	1.128	0.993	0.988	1.031	1.098	1.141	0.963
	2nd	S	1.000	1.000	1.000	1.000	1.000	1.000	1.000	1.000
	3rd	L	0.933	1.075	0.868	1.007	1.028	0.901	1.067	0.844
2013	1st	M	1.050	1.180	0.890	0.992	1.022	1.035	1.189	0.870
	2nd	S	1.000	1.000	1.000	1.000	1.000	1.000	1.000	1.000
	3rd	L	0.936	1.081	0.866	0.998	1.027	0.914	1.083	0.844
2014	1st	M	1.059	1.113	0.951	0.991	1.021	1.046	1.123	0.932
	2nd	L	1.014	1.071	0.947	0.988	1.051	0.977	1.084	0.901
	3rd	S	1.000	1.000	1.000	1.000	1.000	1.000	1.000	1.000
2015	1st	M	1.068	1.183	0.902	1.009	0.982	1.078	1.173	0.919
	2nd	L	1.015	1.065	0.953	0.986	1.022	1.007	1.080	0.932
	3rd	S	1.000	1.000	1.000	1.000	1.000	1.000	1.000	1.000
2016	1st	M	1.011	1.065	0.950	0.988	1.029	0.995	1.078	0.923
	2nd	S	1.000	1.000	1.000	1.000	1.000	1.000	1.000	1.000
	3rd	L	0.963	0.951	1.013	0.984	1.044	0.937	0.966	0.970
2017	1st	M	1.027	1.266	0.812	0.993	1.010	1.025	1.275	0.804
	2nd	L	1.004	1.145	0.877	1.015	1.018	0.971	1.128	0.861
	3rd	S	1.000	1.000	1.000	1.000	1.000	1.000	1.000	1.000
2018	1st	L	1.012	1.313	0.770	0.996	1.040	0.977	1.318	0.741
	2nd	S	1.000	1.000	1.000	1.000	1.000	1.000	1.000	1.000
	3rd	M	0.999	1.515	0.660	0.987	1.022	0.990	1.535	0.645
2019	1st	L	1.030	1.191	0.865	0.993	1.042	0.996	1.200	0.830
	2nd	S	1.000	1.000	1.000	1.000	1.000	1.000	1.000	1.000
	3rd	M	0.983	1.414	0.695	1.001	1.006	0.976	1.412	0.691
2020	1st	L	1.134	1.159	0.978	0.994	1.028	1.109	1.166	0.951
	2nd	M	1.093	1.399	0.781	1.004	1.000	1.090	1.394	0.781
	3rd	S	1.000	1.000	1.000	1.000	1.000	1.000	1.000	1.000
2021	1st	M	1.060	1.374	0.772	1.046	0.984	1.030	1.313	0.784
	2nd	L	1.046	1.213	0.862	1.026	0.996	1.023	1.182	0.865
	3rd	S	1.000	1.000	1.000	1.000	1.000	1.000	1.000	1.000

*CMg_m_*: Multi Group Cost Malmquist Index; *OESg*: Group Overall Efficiency Spread; *CFg_m_*: Multi Group Cost Frontier Gap; *AEg*: Group Allocative Efficiency; *PEg_m_*: Multi Group Price Effect; *Mg_m_*: Multi Group Malmquist Index; *TESg*: Group Technical Efficiency Spread; *TFg_m_*: Multi Group Technical Frontier Gap; S: Small size (<200 beds); M: Medium size (≥200–≤400 beds); L: Large size (>400 beds); Reference: Small size.

**Table 6:** Ranking of groups of hospitals per RHA, according to *CMg_m_* and components during 2009–2021.

Year	Ranking of Groups		*CMg_m_*	*OESg*	*CFg_m_*	*AEg*	*PEg_m_*	*Mg_m_*	*TESg*	*TFg_m_*
2009	1st	*5th RHA*	1.429	1.121	1.274	0.994	1.018	1.412	1.128	1.252
	2nd	*3rd RHA*	1.397	1.082	1.291	1.012	0.986	1.400	1.069	1.310
	3rd	*6th RHA*	1.310	0.935	1.401	0.979	1.010	1.325	0.955	1.387
	4th	*4th RHA*	1.235	1.150	1.074	0.998	0.990	1.250	1.152	1.085
	5th	*7th RHA*	1.125	1.149	0.979	1.007	1.013	1.103	1.141	0.967
	6th	*1st RHA*	1.000	1.000	1.000	1.000	1.000	1.000	1.000	1.000
	7th	*2nd RHA*	0.958	0.986	0.972	0.990	1.004	0.964	0.996	0.968
2010	1st	*6th RHA*	1.355	1.044	1.298	1.004	0.980	1.376	1.039	1.324
	2nd	*3rd RHA*	1.347	1.181	1.141	1.007	0.980	1.365	1.173	1.164
	3rd	*5th RHA*	1.311	1.152	1.138	0.990	1.007	1.315	1.163	1.130
	4th	*4th RHA*	1.251	1.185	1.056	0.991	1.007	1.254	1.196	1.049
	5th	*7th RHA*	1.235	1.235	1.000	1.020	1.000	1.211	1.211	1.000
	6th	*2nd RHA*	1.110	0.993	1.118	0.977	1.008	1.128	1.016	1.110
	7th	*1st RHA*	1.000	1.000	1.000	1.000	1.000	1.000	1.000	1.000
2011	1st	*3rd RHA*	1.362	1.136	1.199	0.997	1.029	1.327	1.140	1.165
	2nd	*5th RHA*	1.325	1.224	1.083	1.013	1.007	1.300	1.209	1.075
	3rd	*6th RHA*	1.252	1.048	1.194	0.988	1.027	1.234	1.061	1.163
	4th	*4th RHA*	1.239	1.149	1.078	1.005	1.007	1.224	1.144	1.070
	5th	*7th RHA*	1.172	1.268	0.924	1.015	1.013	1.140	1.250	0.912
	6th	*2nd RHA*	1.060	0.928	1.143	0.979	1.010	1.073	0.948	1.132
	7th	*1st RHA*	1.000	1.000	1.000	1.000	1.000	1.000	1.000	1.000
2012	1st	*5th RHA*	1.322	1.150	1.150	1.003	1.007	1.309	1.146	1.142
	2nd	*3rd RHA*	1.233	1.029	1.198	0.975	1.051	1.203	1.055	1.140
	3rd	*6th RHA*	1.187	0.959	1.237	1.004	1.001	1.181	0.956	1.236
	4th	*7th RHA*	1.164	1.245	0.935	1.013	1.003	1.146	1.229	0.932
	5th	*4th RHA*	1.163	1.083	1.073	0.997	0.997	1.169	1.086	1.076
	6th	*2nd RHA*	1.037	0.877	1.183	0.999	1.008	1.030	0.878	1.174
	7th	*1st RHA*	1.000	1.000	1.000	1.000	1.000	1.000	1.000	1.000
2013	1st	*5th RHA*	1.233	1.068	1.155	1.005	1.001	1.226	1.062	1.154
	2nd	*3rd RHA*	1.219	0.981	1.244	0.991	1.021	1.205	0.989	1.218
	3rd	*6th RHA*	1.164	0.960	1.213	0.983	1.009	1.173	0.976	1.201
	4th	*4th RHA*	1.156	1.073	1.077	1.024	0.987	1.144	1.049	1.091
	5th	*7th RHA*	1.137	1.163	0.978	1.019	1.001	1.115	1.142	0.976
	6th	*1st RHA*	1.000	1.000	1.000	1.000	1.000	1.000	1.000	1.000
	7th	*2nd RHA*	0.995	0.915	1.087	0.999	1.004	0.992	0.917	1.083
2014	1st	*3rd RHA*	1.150	1.065	1.080	0.997	1.015	1.136	1.068	1.064
	2nd	*7th RHA*	1.101	1.094	1.007	1.040	1.014	1.044	1.052	0.993
	3rd	*6th RHA*	1.092	0.991	1.102	0.993	1.022	1.077	0.998	1.078
	4th	*5th RHA*	1.073	1.087	0.987	1.018	1.004	1.051	1.069	0.983
	5th	*4th RHA*	1.051	1.121	0.938	1.030	0.991	1.030	1.088	0.947
	6th	*1st RHA*	1.000	1.000	1.000	1.000	1.000	1.000	1.000	1.000
	7th	*2nd RHA*	0.922	0.947	0.974	1.028	0.983	0.912	0.921	0.991
2015	1st	*5th RHA*	1.101	1.098	1.002	1.020	0.994	1.086	1.077	1.008
	2nd	*3rd RHA*	1.095	1.090	1.004	0.991	1.018	1.086	1.100	0.987
	3rd	*7th RHA*	1.093	1.094	0.999	1.028	1.002	1.061	1.064	0.997
	4th	*6th RHA*	1.046	1.017	1.029	1.009	0.993	1.044	1.008	1.036
	5th	*4th RHA*	1.031	1.133	0.910	1.018	0.996	1.016	1.113	0.914
	6th	*1st RHA*	1.000	1.000	1.000	1.000	1.000	1.000	1.000	1.000
	7th	*2nd RHA*	0.969	0.882	1.098	1.002	1.001	0.966	0.881	1.097
2016	1st	*7th RHA*	1.301	1.060	1.227	1.018	1.019	1.254	1.041	1.205
	2nd	*6th RHA*	1.266	0.994	1.273	0.966	1.015	1.291	1.029	1.254
	3rd	*5th RHA*	1.242	1.091	1.138	1.013	1.014	1.209	1.077	1.122
	4th	*3rd RHA*	1.226	1.072	1.144	0.970	1.026	1.232	1.105	1.116
	5th	*4th RHA*	1.159	1.120	1.034	1.007	1.006	1.144	1.113	1.028
	6th	*2nd RHA*	1.077	0.967	1.114	1.015	0.997	1.065	0.953	1.117
	7th	*1st RHA*	1.000	1.000	1.000	1.000	1.000	1.000	1.000	1.000
2017	1st	*7th RHA*	1.254	1.063	1.180	1.023	1.009	1.216	1.040	1.170
	2nd	*6th RHA*	1.163	0.919	1.267	0.960	1.021	1.187	0.957	1.240
	3rd	*3rd RHA*	1.105	1.061	1.041	0.981	1.011	1.113	1.082	1.029
	4th	*5th RHA*	1.092	1.065	1.026	1.007	1.001	1.084	1.058	1.025
	5th	*4th RHA*	1.047	1.123	0.933	0.998	1.010	1.039	1.126	0.923
	6th	*1st RHA*	1.000	1.000	1.000	1.000	1.000	1.000	1.000	1.000
	7th	*2nd RHA*	0.973	0.813	1.196	0.998	1.001	0.973	0.815	1.195
2018	1st	*7th RHA*	1.282	1.045	1.226	1.032	1.008	1.232	1.013	1.216
	2nd	*3rd RHA*	1.179	1.083	1.088	0.981	1.012	1.188	1.104	1.076
	3rd	*5th RHA*	1.175	1.125	1.044	1.007	1.004	1.162	1.117	1.040
	4th	*6th RHA*	1.171	0.933	1.255	0.977	1.020	1.176	0.955	1.231
	5th	*4th RHA*	1.111	1.212	0.917	1.015	0.996	1.099	1.194	0.920
	6th	*2nd RHA*	1.031	0.724	1.424	0.953	1.043	1.037	0.760	1.365
	7th	*1st RHA*	1.000	1.000	1.000	1.000	1.000	1.000	1.000	1.000
2019	1st	*7th RHA*	1.301	1.027	1.268	1.002	1.028	1.263	1.024	1.233
	2nd	*3rd RHA*	1.181	1.090	1.083	0.998	1.014	1.166	1.092	1.068
	3rd	*5th RHA*	1.169	1.173	0.997	1.005	1.010	1.151	1.167	0.987
	4th	*6th RHA*	1.168	0.986	1.184	0.984	1.006	1.179	1.002	1.177
	5th	*4th RHA*	1.106	1.232	0.897	1.019	0.986	1.100	1.209	0.910
	6th	*2nd RHA*	1.084	0.873	1.242	0.952	1.036	1.100	0.918	1.198
	7th	*1st RHA*	1.000	1.000	1.000	1.000	1.000	1.000	1.000	1.000
2020	1st	*7th RHA*	1.342	1.123	1.195	1.006	1.002	1.332	1.116	1.193
	2nd	*2nd RHA*	1.183	1.022	1.157	0.956	1.006	1.230	1.069	1.150
	3rd	*6th RHA*	1.172	0.968	1.210	0.998	0.974	1.206	0.971	1.242
	4th	*5th RHA*	1.167	1.125	1.038	0.990	0.990	1.190	1.136	1.048
	5th	*4th RHA*	1.124	1.217	0.923	1.005	0.984	1.137	1.212	0.938
	6th	*3rd RHA*	1.120	1.131	0.991	0.998	1.002	1.119	1.133	0.988
	7th	*1st RHA*	1.000	1.000	1.000	1.000	1.000	1.000	1.000	1.000
2021	1st	*3rd RHA*	1.523	1.157	1.316	0.993	0.999	1.535	1.165	1.317
	2nd	*7th RHA*	1.494	0.996	1.500	0.967	1.016	1.520	1.030	1.476
	3rd	*2nd RHA*	1.292	0.861	1.500	0.869	1.083	1.372	0.991	1.384
	4th	*4th RHA*	1.274	1.038	1.227	0.965	1.060	1.246	1.076	1.158
	5th	*6th RHA*	1.272	0.926	1.374	0.983	0.983	1.317	0.943	1.397
	6th	*5th RHA*	1.266	1.022	1.238	0.951	1.012	1.315	1.075	1.224
	7th	*1st RHA*	1.000	1.000	1.000	1.000	1.000	1.000	1.000	1.000

*CMg_m_*: Multi Group Cost Malmquist Index; *OESg*: Group Overall Efficiency Spread; *CFg_m_*: Multi Group Cost Frontier Gap; *AEg*: Group Allocative Efficiency; *PEg_m_*: Multi Group Price Effect; *Mg_m_*: Multi Group Malmquist Index; *TESg*: Group Technical Efficiency Spread; *TFg_m_*: Multi Group Technical Frontier Gap; RHA: Regional Health Authority; Reference: 1^st^ RHA.

## Data Availability

Raw data were retracted from Ministry of Health of Greece databases esy.net and BI forms after appropriate authorization since they are not publicly available. Cleared datasets are available from the authors upon request and after Ministry of Health of Greece approval.

## Abbreviations

*AE*	Allocative Efficiency Index
*AEC*	Allocative Efficiency Change Index
*AEg*	Group Allocative Efficiency Index
*CE*	Cost Efficiency
*CFg*	Group Cost Frontier Gap Index
*CFg_m_*	Multi group Cost Frontier Gap Index
*CM*	Cost Malmquist Index
*CMg*	Group Cost Malmquist Index
*CMg_m_*	Multi Group Cost Malmquist Index
*CTC*	Cost Technical Change Index
*DEA*	Data Envelopment Analysis
*DMU*	Decision-Making Unit
*ICU*	Intensive Care Unit
*L*	Large-sized hospitals >400 beds
*M*	Medium-sized hospitals >200–<400 beds
*MΙ*	Malmquist Index
*Mg*	Group Malmquist Index
*Mg_m_*	Multi Group Malmquist Index
*MoH*	Ministry of Health
*NHS*	National Health System
*OE*	Overall Efficiency Index
*OEC*	Overall Efficiency Change Index
*OESg*	Group Overall Efficiency Spread Index
*PE*	Price Effect Index
*PEg*	Group Price Effect Index
*PEg_m_*	Multi Group Price Effect Index
*PTE*	Pure Technical Efficiency Index
*RHA*	Regional Health Authority
*S*	Small-sized hospitals < 200 beds
*SE*	Scale Efficiency Index
*TC*	Technical Change Index
*TE*	Technical Efficiency Index
*TEC*	Technical Efficiency Change Index
*TESg*	Group Technical Efficiency Spread Index
*TFg*	Group Technical Frontier Gap Index
*TFg_m_*	Multi Group Technical Frontier Gap Index

## Appendix A

**Table A1:** All 109 included Greek public hospitals grouped according to bed capacity and RHA.

DMU		BC	DMU		BC
1^st^ RHA of *Attiki*			4^th^ RHA of *Macedonia and Thrace (cont.)*		
1	Spiliopouleio Hospital “*Agia Eleni*”	S	9	G.H. of Xanthi	M
2	G.H. of Athens “*Elpis*”	S	10	G.H. of Kilkis	M
3	G.H. “*Pammakaristos*”	S	11	G.H. of Thessaloniki “*Ippokrateio*”	L
4	Children’s Hospital in Penteli	S	12	University G.H. of Alexandroupoli	L
5	G.H. of Nea Ionia “*Konstadopouleio*”	M	13	University G.H. of Thessaloniki “*AHEPA*”	L
6	Children’s Hospital “*P. & A. Kyriakou*”	M	**5^th^ RHA of *Thessaly and Sterea Ellada***		
7	G.H. of Attica “*KAT*”	L	1	G.H. of Amfissa	S
8	G.H. of Athens “*Sotiria*”	L	2	G.H. of Thebes	S
9	G.H. of Athens “*Alexandra*”	L	3	G.H. of Karpenisi	S
10	Merger: G.H. of Attica “*Sismanogleio* and G.H. of Melissia “*Amalia Fleming*”	L	4	G.H. of Levadia	S
11	G.H. of Athens “*Ippokrateio*”	L	5	G.H. of Chalkida	S
12	G.H. of Athens “*Evangelismos*”	L	6	G.H./H.C. of Kymi “*G. Papanikolaou”*	S
13	G.H. of Athens “*Korgialeneio–Benakeio Hellenic Red Cross*”	L	7	G.H.–H.C. of Karystos “*Diokleion*”	S
14	Children’s Hospital “*Agia Sofia*”	L	8	G.H. of Volos “*Achillopouleio*”	M
15	G.H. of Athens “*Georgios Gennimatas*”	L	9	G.H. of Trikala	M
16	G.H. of Athens “*Laiko*”	L	10	G.H. of Karditsa	M
17	G.H. “*Elena Venizelou*”	L	11	G.H. of Larissa “*Koutlibaneio–Triantaphylleio*”	M
**2^nd^ RHA of *Piraeus and Aegean***			12	G.H. of Lamia	M
1	G.H./H.C. of Ikaria	S	13	University G.H. of Larissa	L
2	G.H. of Samos “*Agios Pandeleimon*”	S	**6^th^ RHA of *Peloponnese, Ionian Islands, Epirus and Western Greece***		
3	G.H. of Syros “*Vardakeio & Proio*”	S	1	G.H./H.C. of Lixouri “*Mantzavinateio*”	S
4	G.H./H.C. of Kalymnos “*Vouvaleio*”	S	2	G.H. of Kyparissia	S
5	G.H./H.C. of Kythira “*Triphylleio*”	S	3	G.H. of Kefalonia	S
6	G.H./H.C. of Kos “*Ippokrateion*”	S	4	G.H. of East Achaia–Kalavryta branch	S
7	G.H./H.C. of Limnos	S	5	G.H. of Lefkada	S
8	G.H. of Chios “*Skylitseio*”	S	6	G.H. of Zante “*Agios Dionysios*”	S
9	G.H./H.C. of Naxos	S	7	G.H. of Preveza	S
10	G.H. of Elefsina “*Thriasio*”	M	8	G.H. of East Achaia–Aigio branch	S
11	G.H. of Mytilini “*Vostaneio*”	M	9	G.H. of Lakonia–Sparta branch	S
12	G.H. of Voula “*Asklepieio*”	M	10	G.H./H.C. of Filiates	S
13	G.H. of Rhodes “*Andreas G. Papandreou*”	M	11	Children’s G.H. of Patra “*Karamandaneio*”	S
14	Merger: G.H. of Nikaia “*Agios Panteleimon*” and G.H. of West Attica “*Agia Varvara*”	L	12	G.H. of Heleia–Pyrgos branch	S
15	G.H. of Piraeus “*Tzaneio*”	L	13	G.H. of Aetolia–Acarnania–Agrinio branch	S
16	University G.H. “*Attikon*”	L	14	G.H. of Argolida–Nafplio branch	S
**3^rd^ RHA of *Macedonia***			15	G.H. of Heleia–Amaliada branch	S
1	G.H. of Kozani “*Mamatseio*”	S	16	G.H. of Corinth	S
2	G.H. of Grevena	S	17	G.H./H.C. of Krestena	S
3	G.H. of Pella–Giannitsa branch	S	18	G.H. of Argolida–Argos branch	S
4	G.H. of Pella–Edessa branch	S	19	G.H. of Aetolia–Acarnania–Messolongi branch	S
5	G.H. of Imathia–Naousa branch	S	20	G.H. of Lakonia–Molaoi branch	S
6	G.H. of Florina “*Eleni T. Dimitriou*”	S	21	Panarcadic G.H. of Tripoli “*Evangelistria*”	M
7	G.H. of Thessaloniki “*Agios Dimitrios*”	S	22	G.H. of Arta	M
8	G.H. of Imathia–Veroia branch	S	23	G.H. of Ioannina “*G. Chatzikostas*”	M
9	G.H. of Katerini	S	24	G.H. of Kalamata	M
10	G.H. of Kastoria	S	25	G.H. of Corfu “*Agia Eirini*”	M
11	G.H. of Thessaloniki *“G. Gennimatas*”	M	26	Merger: G.H. of Patra “*Agios Andreas*” and Hospital of Thoracic Diseases of Patra “*Agios Loukas*”	M
12	G.H. of Ptolemaida “*Bodosakeio*”	M	27	University G.H. of Patra “*Panagia Voitheia*”	L
13	G.H. of Thessaloniki “*Papageorgiou*”	L	28	University G.H. of Ioannina	L
14	G.H. of Thessaloniki “*G. Papanikolaou*”	L	**7^th^ RHA of *Crete***		
**4^th^ RHA of *Macedonia and Thrace***			1	G.H. of Lasithi–Siteia branch	S
1	G.H. of Chalkidiki	S	2	G.H. of Agios Nikolaos	S
2	G.H./H.C. of Goumenissa	S	3	G.H. of Lasithi–Neapoli branch	S
3	G.H. of Didimoteicho	S	4	G.H./H.C. of Ierapetra	S
4	G.H. of Serres	M	5	G.H. of Rethymno	M
5	G.H. of Thessaloniki “*Agios Pavlos*”	M	6	G.H. of Chania “*Agios Georgios*”	L
6	G.H. of Komotini “*Sismanogleio*”	M	7	University G.H. of Herakleio	L
7	G.H. of Kavala	M	8	G.H. of Herakleio “*Venizeleio–Pananeio*”	L
8	G.H. of Drama	M			

RHA: Regional Health Authority; DMU: decision-making unit; BC: bed capacity; G.H.: general hospital; H.C.: health centre; S: small size (<200 beds); M: medium size (≥200–≤400 beds); L: large size (>400 beds).

**Table A2:** Cost productivity comparison of groups of hospitals per bed capacity with *CMg_m_* and components during 2009–2021.

Year	Group A–Group B	*CMg_m_*	*OESg*	*CFg_m_*	*AEg*	*PEg_m_*	*Mg_m_*	*TESg*	*TFg_m_*
2009	S–M	1.043	1.364	0.765	1.049	0.957	1.040	1.300	0.800
	S–L	0.983	1.354	0.726	1.055	0.982	0.949	1.284	0.739
	M–L	0.942	0.993	0.948	1.006	1.026	0.913	0.987	0.924
2010	S–M	0.995	1.147	0.867	1.015	0.992	0.988	1.131	0.874
	S–L	0.967	1.051	0.920	1.000	1.048	0.923	1.051	0.878
	M–L	0.972	0.916	1.061	0.986	1.056	0.934	0.930	1.005
2011	S–M	1.083	1.116	0.970	1.012	1.016	1.053	1.103	0.955
	S–L	0.959	1.076	0.891	1.014	1.050	0.901	1.061	0.849
	M–L	0.886	0.964	0.919	1.002	1.034	0.855	0.962	0.889
2012	S–M	1.119	1.128	0.993	0.988	1.031	1.098	1.141	0.963
	S–L	0.933	1.075	0.868	1.007	1.028	0.901	1.067	0.844
	M–L	0.834	0.954	0.874	1.019	0.997	0.821	0.935	0.877
2013	S–M	1.050	1.180	0.890	0.992	1.022	1.035	1.189	0.870
	S–L	0.936	1.081	0.866	0.998	1.027	0.914	1.083	0.844
	M–L	0.891	0.916	0.974	1.005	1.004	0.883	0.911	0.969
2014	S–M	1.059	1.113	0.951	0.991	1.021	1.046	1.123	0.932
	S–L	1.014	1.071	0.947	0.988	1.051	0.977	1.084	0.901
	M–L	0.958	0.962	0.995	0.996	1.029	0.934	0.966	0.967
2015	S–M	1.068	1.183	0.902	1.009	0.982	1.078	1.173	0.919
	S–L	1.015	1.065	0.953	0.986	1.022	1.007	1.080	0.932
	M–L	0.950	0.900	1.056	0.978	1.040	0.934	0.920	1.015
2016	S–M	1.011	1.065	0.950	0.988	1.029	0.995	1.078	0.923
	S–L	0.963	0.951	1.013	0.984	1.044	0.937	0.966	0.970
	M–L	0.952	0.893	1.067	0.996	1.015	0.942	0.896	1.051
2017	S–M	1.027	1.266	0.812	0.993	1.010	1.025	1.275	0.804
	S–L	1.004	1.145	0.877	1.015	1.018	0.971	1.128	0.861
	M–L	0.977	0.905	1.080	1.023	1.008	0.948	0.885	1.071
2018	S–M	0.999	1.515	0.660	0.987	1.022	0.990	1.535	0.645
	S–L	1.012	1.313	0.770	0.996	1.040	0.977	1.318	0.741
	M–L	1.013	0.867	1.168	1.009	1.017	0.987	0.859	1.149
2019	S–M	0.983	1.414	0.695	1.001	1.006	0.976	1.412	0.691
	S–L	1.030	1.191	0.865	0.993	1.042	0.996	1.200	0.830
	M–L	1.048	0.842	1.244	0.992	1.036	1.020	0.850	1.201
2020	S–M	1.093	1.399	0.781	1.004	1.000	1.090	1.394	0.781
	S–L	1.134	1.159	0.978	0.994	1.028	1.109	1.166	0.951
	M–L	1.037	0.828	1.252	0.991	1.029	1.018	0.836	1.217
2021	S–M	1.060	1.374	0.772	1.046	0.984	1.030	1.313	0.784
	S–L	1.046	1.213	0.862	1.026	0.996	1.023	1.182	0.865
	M–L	0.987	0.883	1.117	0.981	1.012	0.993	0.900	1.104

*CMg_m_*: Multi Group Cost Malmquist Index; *OESg*: Group Overall Efficiency Spread; *CFg_m_*: Multi Group Cost Frontier Gap; *AEg*: Group Allocative Efficiency; *PEg_m_*: Multi Group Price Effect; *Mg_m_*: Multi Group Malmquist Index; *TESg*: Group Technical Efficiency Spread; *TFg_m_*: Multi Group Technical Frontier Gap; S: Small size (<200 beds); M: Medium size (≥200–≤400 beds); L: Large size (>400 beds).

**Table A3:** Cost productivity comparison of groups of hospitals per RHA with *CMg_m_* and components during 2009–2021.

Year	Group A–Group B	1– 2	1– 3	1– 4	1– 5	1– 6	1– 7	2– 3	2– 4	2– 5	2– 6	2– 7	3– 4	3– 5	3– 6	3– 7	4– 5	4– 6	4– 7	5– 6	5– 7	6– 7
2009	*CMg_m_*	0.96	1.40	1.24	1.43	1.31	1.13	1.46	1.29	1.49	1.37	1.17	0.88	1.02	0.94	0.81	1.16	1.06	0.91	0.92	0.79	0.86
	*OESg*	0.99	1.08	1.15	1.12	0.94	1.15	1.10	1.17	1.14	0.95	1.16	1.06	1.04	0.86	1.06	0.98	0.81	1.00	0.83	1.02	1.23
	*CFg_m_*	0.97	1.29	1.07	1.27	1.40	0.98	1.33	1.11	1.31	1.44	1.01	0.83	0.99	1.08	0.76	1.19	1.30	0.91	1.10	0.77	0.70
	*AEg*	0.99	1.01	1.00	0.99	0.98	1.01	1.02	1.01	1.00	0.99	1.02	0.99	0.98	0.97	0.99	1.00	0.98	1.01	0.98	1.01	1.03
	*PEg_m_*	1.00	0.99	0.99	1.02	1.01	1.01	0.98	0.99	1.01	1.01	1.01	1.00	1.03	1.02	1.03	1.03	1.02	1.02	0.99	1.00	1.00
	*Mg_m_*	0.96	1.40	1.25	1.41	1.33	1.10	1.45	1.30	1.47	1.37	1.14	0.89	1.01	0.95	0.79	1.13	1.06	0.88	0.94	0.78	0.83
	*TESg*	1.00	1.07	1.15	1.13	0.96	1.14	1.07	1.16	1.13	0.96	1.15	1.08	1.06	0.89	1.07	0.98	0.83	0.99	0.85	1.01	1.19
	*TFg_m_*	0.97	1.31	1.09	1.25	1.39	0.97	1.35	1.12	1.29	1.43	1.00	0.83	0.96	1.06	0.74	1.15	1.28	0.89	1.11	0.77	0.70
2010	*CMg_m_*	1.11	1.35	1.25	1.31	1.35	1.24	1.21	1.13	1.18	1.22	1.11	0.93	0.97	1.01	0.92	1.05	1.08	0.99	1.03	0.94	0.91
	*OESg*	0.99	1.18	1.19	1.15	1.04	1.24	1.19	1.19	1.16	1.05	1.24	1.00	0.98	0.88	1.05	0.97	0.88	1.04	0.91	1.07	1.18
	*CFg_m_*	1.12	1.14	1.06	1.14	1.30	1.00	1.02	0.94	1.02	1.16	0.89	0.93	1.00	1.14	0.88	1.08	1.23	0.95	1.14	0.88	0.77
	*AEg*	0.98	1.01	0.99	0.99	1.00	1.02	1.03	1.01	1.01	1.03	1.04	0.98	0.98	1.00	1.01	1.00	1.01	1.03	1.01	1.03	1.02
	*PEg_m_*	1.01	0.98	1.01	1.01	0.98	1.00	0.97	1.00	1.00	0.97	0.99	1.03	1.03	1.00	1.02	1.00	0.97	0.99	0.97	0.99	1.02
	*Mg_m_*	1.13	1.37	1.25	1.31	1.38	1.21	1.21	1.11	1.17	1.22	1.07	0.92	0.96	1.01	0.89	1.05	1.10	0.97	1.05	0.92	0.88
	*TESg*	1.02	1.17	1.20	1.16	1.04	1.21	1.15	1.18	1.14	1.02	1.19	1.02	0.99	0.89	1.03	0.97	0.87	1.01	0.89	1.04	1.17
	*TFg_m_*	1.11	1.16	1.05	1.13	1.32	1.00	1.05	0.95	1.02	1.19	0.90	0.90	0.97	1.14	0.86	1.08	1.26	0.95	1.17	0.88	0.76
2011	*CMg_m_*	1.06	1.36	1.24	1.33	1.25	1.17	1.28	1.17	1.25	1.18	1.11	0.91	0.97	0.92	0.86	1.07	1.01	0.95	0.94	0.88	0.94
	*OESg*	0.93	1.14	1.15	1.22	1.05	1.27	1.22	1.24	1.32	1.13	1.37	1.01	1.08	0.92	1.12	1.06	0.91	1.10	0.86	1.04	1.21
	*CFg_m_*	1.14	1.20	1.08	1.08	1.19	0.92	1.05	0.94	0.95	1.04	0.81	0.90	0.90	1.00	0.77	1.00	1.11	0.86	1.10	0.85	0.77
	*AEg*	0.98	1.00	1.00	1.01	0.99	1.01	1.02	1.03	1.03	1.01	1.04	1.01	1.02	0.99	1.02	1.01	0.98	1.01	0.98	1.00	1.03
	*PEg_m_*	1.01	1.03	1.01	1.01	1.03	1.01	1.02	1.00	1.00	1.02	1.00	0.98	0.98	1.00	0.98	1.00	1.02	1.01	1.02	1.01	0.99
	*Mg_m_*	1.07	1.33	1.22	1.30	1.23	1.14	1.24	1.14	1.21	1.15	1.06	0.92	0.98	0.93	0.86	1.06	1.01	0.93	0.95	0.88	0.92
	*TESg*	0.95	1.14	1.14	1.21	1.06	1.25	1.20	1.21	1.28	1.12	1.32	1.00	1.06	0.93	1.10	1.06	0.93	1.09	0.88	1.03	1.18
	*TFg_m_*	1.13	1.16	1.07	1.08	1.16	0.91	1.03	0.94	0.95	1.03	0.81	0.92	0.92	1.00	0.78	1.01	1.09	0.85	1.08	0.85	0.78
2012	*CMg_m_*	1.04	1.23	1.16	1.32	1.19	1.16	1.19	1.12	1.27	1.14	1.12	0.94	1.07	0.96	0.94	1.14	1.02	1.00	0.90	0.88	0.98
	*OESg*	0.88	1.03	1.08	1.15	0.96	1.25	1.17	1.24	1.31	1.09	1.42	1.05	1.12	0.93	1.21	1.06	0.89	1.15	0.83	1.08	1.30
	*CFg_m_*	1.18	1.20	1.07	1.15	1.24	0.93	1.01	0.91	0.97	1.05	0.79	0.90	0.96	1.03	0.78	1.07	1.15	0.87	1.08	0.81	0.76
	*AEg*	1.00	0.98	1.00	1.00	1.00	1.01	0.98	1.00	1.00	1.00	1.01	1.02	1.03	1.03	1.04	1.01	1.01	1.02	1.00	1.01	1.01
	*PEg_m_*	1.01	1.05	1.00	1.01	1.00	1.00	1.04	0.99	1.00	0.99	1.00	0.95	0.96	0.95	0.95	1.01	1.00	1.01	0.99	1.00	1.00
	*Mg_m_*	1.03	1.20	1.17	1.31	1.18	1.15	1.17	1.13	1.27	1.15	1.11	0.97	1.09	0.98	0.95	1.12	1.01	0.98	0.90	0.88	0.97
	*TESg*	0.88	1.06	1.09	1.15	0.96	1.23	1.20	1.24	1.31	1.09	1.40	1.03	1.09	0.91	1.16	1.06	0.88	1.13	0.83	1.07	1.29
	*TFg_m_*	1.17	1.14	1.08	1.14	1.24	0.93	0.97	0.92	0.97	1.05	0.79	0.94	1.00	1.08	0.82	1.06	1.15	0.87	1.08	0.82	0.75
2013	*CMg_m_*	1.00	1.22	1.16	1.23	1.16	1.14	1.23	1.16	1.24	1.17	1.14	0.95	1.01	0.95	0.93	1.07	1.01	0.98	0.94	0.92	0.98
	*OESg*	0.92	0.98	1.07	1.07	0.96	1.16	1.07	1.17	1.17	1.05	1.27	1.09	1.09	0.98	1.19	0.99	0.89	1.08	0.90	1.09	1.21
	*CFg_m_*	1.09	1.24	1.08	1.16	1.21	0.98	1.14	0.99	1.06	1.12	0.90	0.87	0.93	0.98	0.79	1.07	1.13	0.91	1.05	0.85	0.81
	*AEg*	1.00	0.99	1.02	1.00	0.98	1.02	0.99	1.02	1.01	0.98	1.02	1.03	1.01	0.99	1.03	0.98	0.96	1.00	0.98	1.01	1.04
	*PEg_m_*	1.00	1.02	0.99	1.00	1.01	1.00	1.02	0.98	1.00	1.01	1.00	0.97	0.98	0.99	0.98	1.01	1.02	1.01	1.01	1.00	0.99
	*Mg_m_*	0.99	1.21	1.14	1.23	1.17	1.11	1.21	1.15	1.24	1.18	1.12	0.95	1.02	0.97	0.93	1.07	1.02	0.97	0.96	0.91	0.95
	*TESg*	0.92	0.99	1.05	1.06	0.98	1.14	1.08	1.14	1.16	1.06	1.25	1.06	1.07	0.99	1.15	1.01	0.93	1.09	0.92	1.07	1.17
	*TFg_m_*	1.08	1.22	1.09	1.15	1.20	0.98	1.12	1.01	1.07	1.11	0.90	0.90	0.95	0.99	0.80	1.06	1.10	0.89	1.04	0.85	0.81
2014	*CMg_m_*	0.92	1.15	1.05	1.07	1.09	1.10	1.25	1.14	1.16	1.18	1.19	0.91	0.93	0.95	0.96	1.02	1.04	1.05	1.02	1.03	1.01
	*OESg*	0.95	1.06	1.12	1.09	0.99	1.09	1.12	1.18	1.15	1.05	1.16	1.05	1.02	0.93	1.03	0.97	0.88	0.98	0.91	1.01	1.10
	*CFg_m_*	0.97	1.08	0.94	0.99	1.10	1.01	1.11	0.96	1.01	1.13	1.03	0.87	0.91	1.02	0.93	1.05	1.18	1.07	1.12	1.02	0.91
	*AEg*	1.03	1.00	1.03	1.02	0.99	1.04	0.97	1.00	0.99	0.97	1.01	1.03	1.02	1.00	1.04	0.99	0.96	1.01	0.98	1.02	1.05
	*PEg_m_*	0.98	1.01	0.99	1.00	1.02	1.01	1.03	1.01	1.02	1.04	1.03	0.98	0.99	1.01	1.00	1.01	1.03	1.02	1.02	1.01	0.99
	*Mg_m_*	0.91	1.14	1.03	1.05	1.08	1.04	1.25	1.13	1.15	1.18	1.14	0.91	0.92	0.95	0.92	1.02	1.05	1.01	1.02	0.99	0.97
	*TESg*	0.92	1.07	1.09	1.07	1.00	1.05	1.16	1.18	1.16	1.08	1.14	1.02	1.00	0.94	0.98	0.98	0.92	0.97	0.93	0.98	1.05
	*TFg_m_*	0.99	1.06	0.95	0.98	1.08	0.99	1.07	0.96	0.99	1.09	1.00	0.89	0.92	1.01	0.93	1.04	1.14	1.05	1.10	1.01	0.92
2015	*CMg_m_*	0.97	1.09	1.03	1.10	1.05	1.09	1.13	1.06	1.14	1.08	1.13	0.94	1.01	0.96	1.00	1.07	1.01	1.06	0.95	0.99	1.05
	*OESg*	0.88	1.09	1.13	1.10	1.02	1.09	1.24	1.28	1.25	1.15	1.24	1.04	1.01	0.93	1.00	0.97	0.90	0.97	0.93	1.00	1.08
	*CFg_m_*	1.10	1.00	0.91	1.00	1.03	1.00	0.91	0.83	0.91	0.94	0.91	0.91	1.00	1.02	1.00	1.10	1.13	1.10	1.03	1.00	0.97
	*AEg*	1.00	0.99	1.02	1.02	1.01	1.03	0.99	1.02	1.02	1.01	1.03	1.03	1.03	1.02	1.04	1.00	0.99	1.01	0.99	1.01	1.02
	*PEg_m_*	1.00	1.02	1.00	0.99	0.99	1.00	1.02	0.99	0.99	0.99	1.00	0.98	0.98	0.98	0.98	1.00	1.00	1.01	1.00	1.01	1.01
	*Mg_m_*	0.97	1.09	1.02	1.09	1.04	1.06	1.12	1.05	1.12	1.08	1.10	0.94	1.00	0.96	0.98	1.07	1.03	1.04	0.96	0.98	1.02
	*TESg*	0.88	1.10	1.11	1.08	1.01	1.06	1.25	1.26	1.22	1.14	1.21	1.01	0.98	0.92	0.97	0.97	0.91	0.96	0.94	0.99	1.06
	*TFg_m_*	1.10	0.99	0.91	1.01	1.04	1.00	0.90	0.83	0.92	0.94	0.91	0.93	1.02	1.05	1.01	1.10	1.13	1.09	1.03	0.99	0.96
2016	*CMg_m_*	1.08	1.23	1.16	1.24	1.27	1.30	1.14	1.08	1.15	1.17	1.21	0.94	1.01	1.03	1.06	1.07	1.09	1.12	1.02	1.05	1.03
	*OESg*	0.97	1.07	1.12	1.09	0.99	1.06	1.11	1.16	1.13	1.03	1.10	1.05	1.02	0.93	0.99	0.97	0.89	0.95	0.91	0.97	1.07
	*CFg_m_*	1.11	1.14	1.03	1.14	1.27	1.23	1.03	0.93	1.02	1.14	1.10	0.90	0.99	1.11	1.07	1.10	1.23	1.19	1.12	1.08	0.96
	*AEg*	1.01	0.97	1.01	1.01	0.97	1.02	0.96	0.99	1.00	0.95	1.00	1.04	1.04	1.00	1.05	1.01	0.96	1.01	0.95	1.01	1.05
	*PEg_m_*	1.00	1.03	1.01	1.01	1.01	1.02	1.03	1.01	1.02	1.02	1.02	0.98	0.99	0.99	0.99	1.01	1.01	1.01	1.00	1.00	1.00
	*Mg_m_*	1.06	1.23	1.14	1.21	1.29	1.25	1.16	1.07	1.14	1.21	1.18	0.93	0.98	1.05	1.02	1.06	1.13	1.10	1.07	1.04	0.97
	*TESg*	0.95	1.10	1.11	1.08	1.03	1.04	1.16	1.17	1.13	1.08	1.09	1.01	0.98	0.93	0.94	0.97	0.93	0.94	0.96	0.97	1.01
	*TFg_m_*	1.12	1.12	1.03	1.12	1.25	1.20	1.00	0.92	1.00	1.12	1.08	0.92	1.01	1.12	1.08	1.09	1.22	1.17	1.12	1.07	0.96
2017	*CMg_m_*	0.97	1.10	1.05	1.09	1.16	1.25	1.14	1.08	1.12	1.20	1.29	0.95	0.99	1.05	1.14	1.04	1.11	1.20	1.07	1.15	1.08
	*OESg*	0.81	1.06	1.12	1.07	0.92	1.06	1.30	1.38	1.31	1.13	1.31	1.06	1.00	0.87	1.00	0.95	0.82	0.95	0.86	1.00	1.16
	*CFg_m_*	1.20	1.04	0.93	1.03	1.27	1.18	0.87	0.78	0.86	1.06	0.99	0.90	0.99	1.22	1.13	1.10	1.36	1.27	1.23	1.15	0.93
	*AEg*	1.00	0.98	1.00	1.01	0.96	1.02	0.98	1.00	1.01	0.96	1.02	1.02	1.03	0.98	1.04	1.01	0.96	1.02	0.95	1.02	1.06
	*PEg_m_*	1.00	1.01	1.01	1.00	1.02	1.01	1.01	1.01	1.00	1.02	1.01	1.00	0.99	1.01	1.00	0.99	1.01	1.00	1.02	1.01	0.99
	*Mg_m_*	0.97	1.11	1.04	1.08	1.19	1.22	1.14	1.07	1.11	1.22	1.25	0.93	0.97	1.07	1.09	1.04	1.14	1.17	1.09	1.12	1.02
	*TESg*	0.81	1.08	1.13	1.06	0.96	1.04	1.33	1.38	1.30	1.17	1.28	1.04	0.98	0.88	0.96	0.94	0.85	0.92	0.90	0.98	1.09
	*TFg_m_*	1.19	1.03	0.92	1.02	1.24	1.17	0.86	0.77	0.86	1.04	0.98	0.90	1.00	1.21	1.14	1.11	1.34	1.27	1.21	1.14	0.94
2018	*CMg_m_*	1.03	1.18	1.11	1.17	1.17	1.28	1.14	1.08	1.14	1.14	1.24	0.94	1.00	0.99	1.09	1.06	1.05	1.15	1.00	1.09	1.10
	*OESg*	0.72	1.08	1.21	1.13	0.93	1.05	1.50	1.67	1.55	1.29	1.44	1.12	1.04	0.86	0.96	0.93	0.77	0.86	0.83	0.93	1.12
	*CFg_m_*	1.42	1.09	0.92	1.04	1.26	1.23	0.76	0.64	0.73	0.88	0.86	0.84	0.96	1.15	1.13	1.14	1.37	1.34	1.20	1.17	0.98
	*AEg*	0.95	0.98	1.02	1.01	0.98	1.03	1.03	1.06	1.06	1.02	1.08	1.03	1.03	1.00	1.05	0.99	0.96	1.02	0.97	1.02	1.06
	*PEg_m_*	1.04	1.01	1.00	1.00	1.02	1.01	0.97	0.96	0.96	0.98	0.97	0.98	0.99	1.01	1.00	1.01	1.02	1.01	1.02	1.00	0.99
	*Mg_m_*	1.04	1.19	1.10	1.16	1.18	1.23	1.15	1.06	1.12	1.13	1.19	0.93	0.98	0.99	1.04	1.06	1.07	1.12	1.01	1.06	1.05
	*TESg*	0.76	1.10	1.19	1.12	0.96	1.01	1.45	1.57	1.47	1.26	1.33	1.08	1.01	0.86	0.92	0.94	0.80	0.85	0.85	0.91	1.06
	*TFg_m_*	1.37	1.08	0.92	1.04	1.23	1.22	0.79	0.67	0.76	0.90	0.89	0.86	0.97	1.14	1.13	1.13	1.34	1.32	1.18	1.17	0.99
2019	*CMg_m_*	1.08	1.18	1.11	1.17	1.17	1.30	1.09	1.02	1.08	1.08	1.20	0.94	0.99	0.99	1.10	1.06	1.06	1.18	1.00	1.11	1.11
	*OESg*	0.87	1.09	1.23	1.17	0.99	1.03	1.25	1.41	1.34	1.13	1.18	1.13	1.08	0.91	0.94	0.95	0.80	0.83	0.84	0.87	1.04
	*CFg_m_*	1.24	1.08	0.90	1.00	1.18	1.27	0.87	0.72	0.80	0.95	1.02	0.83	0.92	1.09	1.17	1.11	1.32	1.41	1.19	1.27	1.07
	*AEg*	0.95	1.00	1.02	1.01	0.98	1.00	1.05	1.07	1.06	1.03	1.05	1.02	1.01	0.99	1.00	0.99	0.97	0.98	0.98	1.00	1.02
	*PEg_m_*	1.04	1.01	0.99	1.01	1.01	1.03	0.98	0.95	0.97	0.97	0.99	0.97	1.00	0.99	1.01	1.02	1.02	1.04	1.00	1.02	1.02
	*Mg_m_*	1.10	1.17	1.10	1.15	1.18	1.26	1.06	1.00	1.05	1.07	1.15	0.94	0.99	1.01	1.08	1.05	1.07	1.15	1.02	1.10	1.07
	*TESg*	0.92	1.09	1.21	1.17	1.00	1.02	1.19	1.32	1.27	1.09	1.12	1.11	1.07	0.92	0.94	0.97	0.83	0.85	0.86	0.88	1.02
	*TFg_m_*	1.20	1.07	0.91	0.99	1.18	1.23	0.89	0.76	0.82	0.98	1.03	0.85	0.92	1.10	1.15	1.08	1.29	1.35	1.19	1.25	1.05
2020	*CMg_m_*	1.18	1.12	1.12	1.17	1.17	1.34	0.95	0.95	0.99	0.99	1.13	1.00	1.04	1.05	1.20	1.04	1.04	1.19	1.00	1.15	1.15
	*OESg*	1.02	1.13	1.22	1.12	0.97	1.12	1.11	1.19	1.10	0.95	1.10	1.08	0.99	0.86	0.99	0.92	0.80	0.92	0.86	1.00	1.16
	*CFg_m_*	1.16	0.99	0.92	1.04	1.21	1.20	0.86	0.80	0.90	1.05	1.03	0.93	1.05	1.22	1.21	1.12	1.31	1.30	1.17	1.15	0.99
	*AEg*	0.96	1.00	1.00	0.99	1.00	1.01	1.04	1.05	1.04	1.04	1.05	1.01	0.99	1.00	1.01	0.99	0.99	1.00	1.01	1.02	1.01
	*PEg_m_*	1.01	1.00	0.98	0.99	0.97	1.00	1.00	0.98	0.98	0.97	1.00	0.98	0.99	0.97	1.00	1.01	0.99	1.02	0.98	1.01	1.03
	*Mg_m_*	1.23	1.12	1.14	1.19	1.21	1.33	0.91	0.92	0.97	0.98	1.08	1.02	1.06	1.08	1.19	1.05	1.06	1.17	1.01	1.12	1.10
	*TESg*	1.07	1.13	1.21	1.14	0.97	1.12	1.06	1.13	1.06	0.91	1.04	1.07	1.00	0.86	0.99	0.94	0.80	0.92	0.85	0.98	1.15
	*TFg_m_*	1.15	0.99	0.94	1.05	1.24	1.19	0.86	0.82	0.91	1.08	1.04	0.95	1.06	1.26	1.21	1.12	1.32	1.27	1.19	1.14	0.96
2021	*CMg_m_*	1.29	1.52	1.27	1.27	1.27	1.49	1.18	0.99	0.98	0.98	1.16	0.84	0.83	0.84	0.98	0.99	1.00	1.17	1.01	1.18	1.17
	*OESg*	0.86	1.16	1.04	1.02	0.93	1.00	1.34	1.20	1.19	1.08	1.16	0.90	0.88	0.80	0.86	0.99	0.89	0.96	0.91	0.97	1.08
	*CFg_m_*	1.50	1.32	1.23	1.24	1.37	1.50	0.88	0.82	0.83	0.92	1.00	0.93	0.94	1.04	1.14	1.01	1.12	1.22	1.11	1.21	1.09
	*AEg*	0.87	0.99	0.96	0.95	0.98	0.97	1.14	1.11	1.09	1.13	1.11	0.97	0.96	0.99	0.97	0.99	1.02	1.00	1.03	1.02	0.98
	*PEg_m_*	1.08	1.00	1.06	1.01	0.98	1.02	0.92	0.98	0.93	0.91	0.94	1.06	1.01	0.98	1.02	0.95	0.93	0.96	0.97	1.00	1.03
	*Mg_m_*	1.37	1.53	1.25	1.32	1.32	1.52	1.12	0.91	0.96	0.96	1.11	0.81	0.86	0.86	0.99	1.06	1.06	1.22	1.00	1.16	1.15
	*TESg*	0.99	1.17	1.08	1.07	0.94	1.03	1.18	1.09	1.08	0.95	1.04	0.92	0.92	0.81	0.88	1.00	0.88	0.96	0.88	0.96	1.09
	*TFg_m_*	1.38	1.32	1.16	1.22	1.40	1.48	0.95	0.84	0.88	1.01	1.07	0.88	0.93	1.06	1.12	1.06	1.21	1.27	1.14	1.21	1.06

*CMg_m_*: Multi Group Cost Malmquist Index; *OESg*: Group Overall Efficiency Spread; *CFg_m_*: Multi Group Cost Frontier Gap; *AEg*: Group Allocative Efficiency; *PEg_m_*: Multi Group Price Effect; *Mg_m_*: Multi Group Malmquist Index; *TESg*: Group Technical Efficiency Spread; *TFg_m_*: Multi Group Technical Frontier Gap; RHA: Regional Health Authority.

## References

[1] European Commission (2010). The Economic Adjustment Programme for Greece.

[2] European Commission (2010). The Economic Adjustment Programme for Greece First Review, Summer 2010.

[3] European Commission (2010). The Economic Adjustment Programme for Greece: Second Review, Autumn 2010.

[4] European Commission (2011). The Economic Adjustment Programme for Greece: Third Review—Winter 2011.

[5] European Commission (2011). The Economic Adjustment Programme for Greece: Fourth Review, Spring 2011.

[6] European Commission (2011). The Economic Adjustment Programme for Greece: Fifth Review, October 2011.

[7] European Commission (2012). The Second Economic Adjustment Programme for Greece.

[8] European Commission (2012). The Second Economic Adjustment Programme for Greece First Review, December 2012.

[9] European Commission (2013). The Second Economic Adjustment Programme for Greece: Second Review—May 2013.

[10] European Commission (2013). The Second Economic Adjustment Programme for Greece: Third Review—July 2013.

[11] European Commission (2014). The Second Economic Adjustment Programme for Greece.

[12] European Commission (2017). The ESM Stability Support Programme: Greece, First & Second Reviews—July 2017: Background Report.

[13] European Commission (2018). Compliance Report ESM Stability Support Program for Greece, Fourth Review, July 2018.

[14] Ladi S., Angelou A., Panagiotatou D. (2022). Regaining Trust: Evidence-Informed Policymaking during the First Phase of the COVID-19 Crisis in Greece. South Eur. Soc. Polit..

[15] Lampropoulou M. (2021). Public Administration in the Era of COVID-19: Policy Responses and Reforms Underway.

[16] Sotiropoulos D.A., Huliaras A., Karadag R. (2021). Greece Report. Sustainable Governance in the Context of the COVID-19 Crisis.

[17] Economou C., Kaitelidou D., Konstantakopoulou O., Vildiridi L.V. Policy Responses for Greece; COVID-19 Health System Response Monitor, European Observatory on Health Systems and Policies, 2020. https://eurohealthobservatory.who.int/monitors/hsrm/hsrm-countries/hsrm/greece/overview/.

[18] Sypsa V., Roussos S., Paraskevis D., Lytras T., Tsiodras S., Hatzakis A. (2021). Effects of Social Distalncing Measures during the First Epidemic Wave of Severe Acute Respiratory Syndrome Infection, Greece. Emerg. Infect. Dis..

[19] Lytras T., Tsiodras S. (2022). Total Patient Load, Regional Disparities and in-Hospital Mortality of Intubated COVID-19 Patients in Greece, from September 2020 to May 2021. Scand. J. Public Health.

[20] Economou C., Kaitelidou D., Karanikolos M., Maresso A. (2017). Greece: Health System Review. Health Syst. Transit. Eur. Obs. Health Syst. Policies.

[21] Economou C., Kaitelidou D., Kentikelenis A., Sissouras A., Maresso A. The Impact of the Financial Crisis on the Health System and Health in Greece: Case Study; World Health Organization, Regional Office for Europe, European Observatory on Health Systems and Policies, 2014; 48p. https://iris.who.int/bitstream/handle/10665/366495/WHO-EURO-2014-6438-46204-66837-eng.pdf?sequence=1&isAllowed=y.

[22] Camanho A.S., Dyson R.G. (2006). Data Envelopment Analysis and Malmquist Indices for Measuring Group Performance. J. Product. Anal..

[23] Thanassoulis E., Shiraz R.K., Maniadakis N. (2015). A Cost Malmquist Productivity Index Capturing Group Performance. Eur. J. Oper. Res..

[24] Maniadakis N., Thanassoulis E. (2000). Assessing Productivity Changes in UK Hospitals Reflecting Technology and Input Prices. Appl. Econ..

[25] Maniadakis N., Thanassoulis E. (2004). A Cost Malmquist Productivity Index. Eur. J. Oper. Res..

[26] Malmquist S. (1953). Index Numbers and Indifference Surfaces. Trab. Estad..

[27] Emrouznejad A., Yang G. (2018). A Survey and Analysis of the First 40 Years of Scholarly Literature in DEA: 1978–2016. Socioecon. Plann. Sci..

[28] Caves D.W., Christensen L.R., Diewert W.E. (1982). The Economic Theory of Index Numbers and the Measurement of Input, Output, and Productivity. Econometrica.

[29] Färe R., Grosskopf S., Lovell C.A.K. (1993). Production Frontiers.

[30] Färe R., Grosskopf S., Russell R.R. (1998). Index Numbers: Essays in Honour of Sten Malmquist.

[31] Färe R., Grosskopf S., Lindgren B., Roos P. (1994). Productivity Developments in Swedish Hospitals: A Malmquist Output Index Approach. Data Envelopment Analysis: Theory, Methodology, and Applications.

[32] Färe R., Grosskopf S., Norris M., Zhang Z. (1994). Productivity Growth, Technical Progress, and Efficiency Change in Industrialized Countries. Am. Econ. Rev..

[33] Asghar N., Rehman H.U., Ali M. (2019). Cost Productivity of Healthcare Systems in OIC’s Member Countries: An Application of Cost Malmquist Total Productivity Index. Rev. Econ. Dev. Stud..

[34] Hosseinzadeh L.F., Jahanshahloo G.R., Akbarian D. (2007). New Insights into Cost Malmquist Productivity Measure. Int. J. Contemp. Math. Sci..

[35] Tohidi G., Razavyan S., Tohidnia S. (2012). A Global Cost Malmquist Productivity Index Using Data Envelopment Analysis. J. Oper. Res. Soc..

[36] Ali M., Mushtaq S., Abdullah M. (2021). A Quest for Growth In Cost Productivity of Agriculture Sector. PalArchs J. Archaeol. Egypt Egyptol..

[37] Baležentis T. (2012). The Cost Malmquist Index Decomposition for Analysis of the Total Factor Productivity Change in Lithuanian Family Farms. Žemės Ūkio Moksl..

[38] Bilotkach V., Gitto S., Jovanović R., Mueller J., Pels E. (2015). Cost-Efficiency Benchmarking of European Air Navigation Service Providers. Transp. Res. Part Policy Pract..

[39] Brzezicki L., Lacka I. (2022). Cost Efficiency of Administrative Service in Public Higher Education in Poland. Sci. Pap. Silesian Univ. Technol. Organ. Manag. Ser..

[40] Cho T. (2020). Cost Metafrontier Approach for Measuring the Malmquist Productivity Index: An Example of Bank Groups Formed after the Financial Reform in Taiwan. Pac. Econ. Rev..

[41] Cho T.-Y., Chen Y.-S. (2021). The Impact of Financial Technology on China’s Banking Industry: An Application of the Metafrontier Cost Malmquist Productivity Index. North Am. J. Econ. Finance.

[42] Cho T.-Y., Wang T.-Y. (2018). Estimations of Cost Metafrontier Malmquist Productivity Index: Using International Tourism Hotels in Taiwan as an Example. Empir. Econ..

[43] Du M., Wang B., Chen Z., Liao L. (2023). Productivity Evaluation of Urban Water Supply Industry in China: A Metafrontier-Biennial Cost Malmquist Productivity Index Approach. Ann. Oper. Res..

[44] Huang M.-Y., Juo J.-C., Fu T. (2015). Metafrontier Cost Malmquist Productivity Index: An Application to Taiwanese and Chinese Commercial Banks. J. Product. Anal..

[45] Liao W.-L., Yao C.-Y., Yang Y.-P., Hung J.C., Yen N.Y., Hui L. (2019). Effect of Fintech on the Cost Malmquist Productivity Index in the China Banking Industry. Frontier Computing.

[46] Maziotis A., Sala-Garrido R., Mocholi-Arce M., Molinos-Senante M. (2021). Changes to The Productivity of Water Companies: Comparison of Fully Private and Concessionary Water Companies. Water Resour. Manag..

[47] Ohene-Asare K., Asare J.K.A., Turkson C. (2019). Dynamic Cost Productivity and Economies of Scale of Ghanaian Insurers. Geneva Pap. Risk Insur.—Issues Pract..

[48] Wang Y.-S., Xie B.-C., Shang L.-F., Li W.-H. (2013). Measures to Improve the Performance of China’s Thermal Power Industry in View of Cost Efficiency. Appl. Energy.

[49] Yang Y.-L., Sheng T.-C., Huang M.-H. (2010). Estimating the Cost Malmquist Productivity Index in the Taiwan Biotech and Biopharmaceutical Industry. Appl. Econ..

[50] Yang Y.-L., Lee J.-Y. (2009). Decomposition of the Cost Growth for the US Forest Product Industries. Appl. Econ. Lett..

[51] Zhu N., Liu Y., Emrouznejad A., Huang Q. (2017). An Allocation Malmquist Index with an Application in the China Securities Industry. Oper. Res..

[52] Zhu Z., Zhang X., Wang Y., Chen X. (2021). Energy Cost Performance of Thermal Power Industry in China Considering Regional Heterogeneity: A Meta-Frontier Cost Malmquist Productivity Decomposition Approach. Sustainability.

[53] Habibpoor M., Alirezaee M., Rashidinia J. (2022). Group Cost Malmquist Productivity Index: A Case Study of Bank Industry. Ind. Manag. J..

[54] Walheer B. (2018). Cost Malmquist Productivity Index: An Output-Specific Approach for Group Comparison. J. Product. Anal..

[55] Walheer B. (2022). Global Malmquist and Cost Malmquist Indexes for Group Comparison. J. Product. Anal..

[56] Farrell M.J. (1957). The Measurement of Productive Efficiency. J. R. Stat. Soc. Ser. Gen..

[57] Charnes A., Cooper W.W., Rhodes E. (1978). Measuring the Efficiency of Decision Making Units. Eur. J. Oper. Res..

[58] Banker R.D., Charnes A., Cooper W.W. (1984). Some Models for Estimating Technical and Scale Inefficiencies in Data Envelopment Analysis. Manag. Sci..

[59] Hollingsworth B., Dawson P.J., Maniadakis N. (1999). Efficiency Measurement of Health Care: A Review of Non-Parametric Methods and Applications. Health Care Manag. Sci..

[60] Katharakis G. (2012). Evaluation of Health Care Units Through Stochastic Processes. Ph.D. Dissertation.

[61] Chilingerian J.A., Sherman H.D., Cooper W.W., Seiford L.M., Zhu J. (2011). Health-Care Applications: From Hospitals to Physicians, from Productive Efficiency to Quality Frontiers. Handbook on Data Envelopment Analysis.

[62] Dlouhý M. (2009). Efficiency and Productivity Analysis in Health Services. Prague Econ. Pap..

[63] Hollingsworth B. (2008). The Measurement of Efficiency and Productivity of Health Care Delivery. Health Econ..

[64] Iparraguirre J.L., Ma R. (2015). Efficiency in the Provision of Social Care for Older People. A Three-Stage Data Envelopment Analysis Using Self-Reported Quality of Life. Socioecon. Plann. Sci..

[65] de Lobo M.S.C., Lins M.P.E. (2010). Epistemic Dialog between Health Services and Operations Research. Pesqui. Oper..

[66] Maniadakis N., Kotsopoulos N., Prezerakos P., Yfantopoulos J. (2009). Health Care Services Performance Measurement: Theory, Methods and Empirical Evidence. Eur. Res. Stud. J..

[67] Mutter R.L., Rosko M.D., Greene W.H., Wilson P.W. (2011). Translating Frontiers Into Practice: Taking the Next Steps Toward Improving Hospital Efficiency. Med. Care Res. Rev..

[68] Katharakis G., Katharaki M., Katostaras T. (2013). SFA vs. DEA for Measuring Healthcare Efficiency: A Systematic Review. Int. J. Stat. Med. Res..

[69] See K.F., Grosskopf S., Valdmanis V.G., Zelenyuk V. (2021). What Do We Know from the Vast Literature on Efficiency and Productivity in Healthcare?: A Systematic Review and Bibliometric Analysis.

[70] Bahadori M., Izadi A.R., Ghardashi F., Ravangard R., Hosseini S.M. (2016). The Evaluation of Hospital Performance in Iran: A Systematic Review Article. Iran. J. Public Health.

[71] Dong S., Zuo Y., Guo S., Li M., Liu X., Li H. (2017). Data Envelopment Analysis for Relative Efficiency Measurement of Chinese Hospitals: A Systematic Review. Res. Health Sci..

[72] Gandhi A.V., Sharma D. (2018). Technical Efficiency of Private Sector Hospitals in India Using Data Envelopment Analysis. Benchmarking Int. J..

[73] Hadji B., Meyer R., Melikeche S., Escalon S., Degoulet P. (2014). Assessing the Relationships Between Hospital Resources and Activities: A Systematic Review. J. Med. Syst..

[74] Kiadaliri A.A., Jafari M., Gerdtham U.-G. (2013). Frontier-Based Techniques in Measuring Hospital Efficiency in Iran: A Systematic Review and Meta-Regression Analysis. BMC Health Serv. Res..

[75] Kounetas K., Papathanassopoulos F. (2013). How Efficient Are Greek Hospitals? A Case Study Using a Double Bootstrap DEA Approach. Eur. J. Health Econ..

[76] Nguyen K.-H., Coelli T. (2009). Quantifying the Effects of Modelling Choices on Hospital Efficiency Measures.

[77] O’Neill L., Rauner M., Heidenberger K., Kraus M. (2008). A Cross-National Comparison and Taxonomy of DEA-Based Hospital Efficiency Studies. Socioecon. Plann. Sci..

[78] Sibbel R., Nagarajah B. (2012). Are privately owned hospitals more efficient? Results of a survey of the international literature. Gesundheitswesen.

[79] Xenos P., Nektarios M., Polyzos N., Yfantopoulos J. (2014). Modern Methods of Hospital Funding, Competition and Financial Incentives. Arch. Hell. Med..

[80] Seiford L.M. (2005). A Cyber-Bibliography for Data Envelopment Analysis (1978–2005).

[81] Flokou A., Aletras V., Niakas D. (2017). A Window-DEA Based Efficiency Evaluation of the Public Hospital Sector in Greece during the 5-Year Economic Crisis. PLoS ONE.

[82] Antunes B.B.P., Bastos L.S.L., Hamacher S., Bozza F.A. (2021). Using Data Envelopment Analysis to Perform Benchmarking in Intensive Care Units. PLoS ONE.

[83] Harrison J.P., Harrison D.A., Harrison L.O. (2022). Does the Efficiency Frontier of Large US Hospitals Provide a Strategy for Future Success?. Int. J. Nurs. Health Care Res..

[84] Hadian S.A., Rezayatmand R., Ketabi S., Shaarbafchizadeh N., Pourghaderi A.R. (2025). Evaluating Hospital Performance with Additive DEA and MPI: The Isfahan University of Medical Science Case Study. BMC Health Serv. Res..

[85] Sun M., Ye Y., Zhang G., Xue Y., Shang X. (2023). Measuring the Efficiency of Public Hospitals: A Multistage Data Envelopment Analysis in Fujian Province, China. Front. Public Health.

[86] Cheng Z., Tao H., Cai M., Lin H., Lin X., Shu Q., Zhang R. (2015). Technical Efficiency and Productivity of Chinese County Hospitals: An Exploratory Study in Henan Province, China. BMJ Open.

[87] Medarević A., Vuković D. (2021). Efficiency and Productivity of Public Hospitals in Serbia Using DEA-Malmquist Model and Tobit Regression Model, 2015–2019. Int. J. Environ. Res. Public. Health.

[88] Piubello Orsini L., Leardini C., Vernizzi S., Campedelli B. (2021). Inefficiency of Public Hospitals: A Multistage Data Envelopment Analysis in an Italian Region. BMC Health Serv. Res..

[89] Polyzos N. (2012). A Three-Year Performance Evaluation of the NHS Hospitals in Greece. Hippokratia.

[90] Vaňková I., Vrabková I. (2014). The Factors Influencing Economic Efficiency of the Hospital Bed Care in Terms of the Regional Allowance Organizations. Rev. Econ. Perspect..

[91] Xenos P., Nektarios M., Constantopoulos A., Yfantopoulos J. (2016). Two-Stage Hospital Efficiency Analysis Including Qualitative Evidence: A Greek Case. J. Hosp. Adm..

